# Quality of Life in Patients With Variant Syndromes of Autoimmune Liver Diseases—A Cross‐Sectional Multicentre Study

**DOI:** 10.1111/liv.70526

**Published:** 2026-02-02

**Authors:** Natalie Uhlenbusch, Romée J. A. L. M. Snijders, Meike Mund, Maciej K. Janik, Piotr Milkiewicz, Bernd Löwe, Claudia Kroll, Joanna Raszeja‐Wyszomirska, Alessio Gerussi, Francesca Bolis, Laura Cristoferi, Pietro Invernizzi, Patricia Kovats, Mária Papp, Lisbet Grønbæk, Henning Grønbæk, Eric T. T. L. Tjwa, Luise Aamann, Henriette Ytting, Vincenzo Ronca, Kathryn Olsen, Ye H. Oo, Adriaan J. van der Meer, João Madaleno, Bernardo Canhão, Bastian Engel, Alejandro Campos‐Murguia, Richard Taubert, Özgür M. Koc, Matthijs Kramer, José A. Willemse, Ansgar W. Lohse, Joost P. H. Drenth, Tom J. G. Gevers, Christoph Schramm

**Affiliations:** ^1^ Department of Psychosomatic Medicine and Psychotherapy University Medical Centre Hamburg‐Eppendorf Hamburg Germany; ^2^ European Reference Network on Hepatological Diseases (ERN RARE‐LIVER) Hamburg Germany; ^3^ Department of Gastroenterology and Hepatology Radboud University Medical Centre Nijmegen the Netherlands; ^4^ Martin Zeitz Centre for Rare Diseases University Medical Centre Hamburg‐Eppendorf Hamburg Germany; ^5^ Department of Hepatology, Transplantology and Internal Medicine Medical University of Warsaw Warsaw Poland; ^6^ Translational Medicine Group Pomeranian Medical University Szczecin Poland; ^7^ Division of Gastroenterology, Center for Autoimmune Liver Diseases, European Reference Network on Hepatological Diseases (ERN RARE‐LIVER) IRCCS Fondazione San Gerardo Dei Tintori Monza Italy; ^8^ Department of Medicine and Surgery University of Milano‐Bicocca Monza Italy; ^9^ Division of Gastroenterology, Department of Internal Medicine, Faculty of Medicine University of Debrecen Debrecen Hungary; ^10^ Kálmán Laki Doctoral School, Faculty of Medicine University of Debrecen Debrecen Hungary; ^11^ Department of Hepatology and Gastroenterology Aarhus University Hospital Aarhus Denmark; ^12^ Department of Medicine Regional Hospital Horsens Horsens Denmark; ^13^ Department of Gastroenterology and Hepatology Hvidovre University Hospital Copenhagen Hvidovre Denmark; ^14^ Department of Clinical Medicine, Faculty of Health Sciences University of Copenhagen Copenhagen Denmark; ^15^ Liver Transplant and Hepatobiliary Unit, Queen Elizabeth Hospital, University Hospital of Birmingham NHS Foundation Trust & Centre for Liver and Gastro Research, NIHR Biomedical Research Centre, Institute of Immunology and Immunotherapy University of Birmingham Birmingham UK; ^16^ Department of Gastroenterology and Hepatology Erasmus University Medical Centre Rotterdam the Netherlands; ^17^ Liver Disease Unit, Department of Internal Medicine Hospitais da Universidade de Coimbra, Unidade Local de Saúde de Coimbra Coimbra Portugal; ^18^ Liver Transplant Unit, Hospitais da Universidade de Coimbra, Unidade Local de Saúde de Coimbra, Faculty of Medicine University of Coimbra Coimbra Portugal; ^19^ Department of Gastroenterology, Hepatology, Infectious Diseases and Endocrinology Hannover Medical School Hannover Germany; ^20^ Department of Gastroenterology and Hepatology MUMC+ Maastricht the Netherlands; ^21^ GROW—School for Oncology and Reproduction Maastricht University Maastricht the Netherlands; ^22^ Dutch Liver Patients Association Amersfoort the Netherlands; ^23^ I. Department of Medicine University Medical Centre Hamburg‐Eppendorf Hamburg Germany; ^24^ Department of Gastroenterology and Hepatology Amsterdam UMC Amsterdam the Netherlands; ^25^ Nutrim School for Nutrition and Translational Research in Metabolism Maastricht University Maastricht the Netherlands

**Keywords:** autoimmune hepatitis, autoimmune liver diseases, cholestatic liver diseases, overlap syndrome, quality of life, variant syndrome

## Abstract

**Background & Aims:**

Primary biliary cholangitis (PBC), primary sclerosing cholangitis (PSC) and autoimmune hepatitis (AIH) go along with reduced health‐related quality of life (HRQOL). Variant syndromes, that is, conditions with features of both PBC/PSC and AIH, are associated with higher clinical complexity and worse prognosis. Studies on HRQOL in patients with variant syndromes are lacking. We aimed to provide large‐scale evidence addressing this gap.

**Methods:**

We included adult patients with clinical diagnoses of autoimmune liver diseases across nine countries in a cross‐sectional study. We descriptively compared demographical, clinical and patient‐reported outcomes between the conditions and investigated whether additional AIH contributes to reduced HRQOL compared to the cholestatic liver disease alone. Further, we explored the role of fatigue, cirrhosis and depression severity regarding HRQOL.

**Results:**

*N* = 1275 patients were included (PBC: *n* = 342, PBC‐AIH: *n* = 160, PSC: *n* = 305, PSC‐AIH: *n* = 121, AIH: *n* = 347). Patients with variant syndromes showed high rates of cirrhosis and increased depressive or anxiety symptoms. Additional AIH was associated with further reduction in physical and mental HRQOL in people with PSC (physical: Δ*R* ^2^ = 0.012, *p* = 0.041; mental: Δ*R* ^2^ = 0.016, *p* = 0.020), but not with PBC (physical: Δ*R* ^2^ = 0.008, *p* = 0.081; mental: Δ*R* ^2^ = 0.001, *p* = 0.609). Physical HRQOL was associated with higher age and fatigue, while mental HRQOL was associated with lower age, fatigue, and depression severity.

**Conclusions:**

Patients with variant syndromes of autoimmune liver diseases show high physical and mental burden, with fatigue as the main contributor. Particularly PSC‐AIH goes along with more severely reduced HRQOL compared to the cholestatic liver disease alone, which is attributable to a higher symptom burden.

## Key Points

- This multicenter cross‐sectional study provides the first large‐scale evaluation of health‐related quality of life (HRQOL) in variant syndromes of autoimmune liver diseases.
- Patients with variant syndromes show substantial impairments in both physical and mental well‐being.
- PSC–AIH is associated with lower HRQOL compared to PSC alone, largely attributable to higher symptom burden.
- In PBC, additional AIH does not significantly contribute to HRQOL beyond the impact of the underlying cholestatic disease.
- Fatigue is the strongest predictor of HRQOL across groups, while depression severity particularly affects mental HRQOL.

**Abbreviations**
AASLD: American association for the study of liver diseases
AIH: autoimmune hepatitis
EASL: European association for the study of the liver
ERN RARE‐LIVER: European reference network for rare liver diseases
GAD‐7: generalised anxiety disorder 7‐item scale
HRQOL: health‐related quality of life
MFIS: modified fatigue impact scale
PBC: primary biliary cholangitis
PHQ‐9: patient health questionnaire 9
PRO: patient‐reported outcomes
PSC: primary sclerosing cholangitis
SF12v2: short form health survey 12—version 2
SSD‐12: somatic symptom disorder b—criteria—scale
SSS‐8: somatic symptom scale

## 1 Introduction

Primary biliary cholangitis (PBC), primary sclerosing cholangitis (PSC) and autoimmune hepatitis (AIH) are distinct diseases, but there are clinical conditions with features of two diseases in the same patient, which is described with the term ‘overlap syndrome’ or ‘variant syndrome’. A PBC‐AIH variant syndrome occurs when features of both PBC and AIH are present in the same individual as seen in 8%–10% of the patients with either PBC or AIH [1, 2]. Equally, a PSC‐AIH variant syndrome involves features of both PSC and AIH presenting in 7%–14% of the patients with PSC and 10% of those with AIH [3]. It is suggested that variant syndromes should be considered the primary cholestatic liver disease with additional hepatitis activity of varying and often age‐dependent degrees [4]. Due to a diverse spectrum of disease manifestations and the lack of a stringent classification system, variant syndromes are often challenging to diagnose. However, recognising a variant syndrome is crucial as additional immunosuppressive treatment may become necessary to significantly improve prognosis. Due to a scarcity of robust primary evidence concerning variant syndromes, which is indicated in the treatment guidelines provided by the American Association for the Study of Liver Diseases (AASLD [5, 6, 7]) and the European Association for the Study of the Liver (EASL [1, 2, 3]), these syndromes remain poorly understood.

Several studies have demonstrated that patients with autoimmune liver diseases have impaired health‐related quality of life (HRQOL) [8, 9, 10, 11, 12, 13]. Due to the chronic nature of autoimmune liver diseases, improving quality of life is a central healthcare aim. In general, a number of variables, such as the course of the disease, comorbidities and possibly also therapies, might have an impact on HRQOL of patients with autoimmune liver disorders. However, some factors have consistently been linked to HRQOL in this population in previous studies. Fatigue has been identified as a key symptom affecting the well‐being of patients with PBC [11, 14, 15], but also affecting a large number of patients with PSC [16]. Moreover, cirrhosis has repeatedly been found to be associated with HRQOL [17, 18, 19]. Depression severity is another significant variable frequently linked to reduced HRQOL in patients with autoimmune liver diseases [9, 15, 17, 20, 21]. In a prospective study on AIH, Janik and colleagues (2019) describe depression as a dominant symptom affecting patients' well‐being, independent of clinical or biochemical disease features. Depression symptoms have also been linked to steroid treatment in patients with AIH [21]. Furthermore, age and gender play important roles in determining HRQOL, which is a consistent finding across various populations and has been confirmed in the context of autoimmune liver diseases [12, 22, 23].

In patients with variant syndromes, quality of life may be even more impaired. Studies indicate that these conditions, particularly PSC‐AIH, have a worse prognosis in terms of disease progression to liver transplantation or death [24, 25, 26]. The increased clinical complexity of variant syndromes may further result in higher diagnostic challenges, a more severe symptom burden, more intensive medical management as well as a significant mental burden. One study indicated that patients with PBC or PSC in addition to AIH show worse HRQOL compared to patients with AIH alone [8]. However, these data relied on a self‐reported diagnosis of autoimmune liver diseases and the subgroups were quite small, particularly with regard to PSC‐AIH. Thus, there is a lack of large‐scale evidence on HRQOL in patients with variant syndromes, and specifically on how additional AIH may affect people with cholestatic liver disease.

To address this knowledge gap, we set out to investigate physical and mental well‐being in a large sample of people with autoimmune liver diseases including patients with variant syndromes. We aimed to (1) explore the differences between variant syndromes and PBC, PSC and AIH alone regarding sociodemographic and clinical outcomes, psychopathology, and HRQOL, (2) investigate whether being affected by a variant syndrome contributes to reduced HRQOL compared to the cholestatic liver disease alone (after controlling for gender and age), and (3) explore the association of fatigue, cirrhosis and depression severity with HRQOL in this population.

## 2 Patients and Methods

### 2.1 Design and Setting

This is a cross‐sectional multicenter study. Twelve university medical centers (including seven liver transplant centers) across nine European countries recruited patients between July 2020 and June 2023. Nine of the participating centers are part of the European Reference Network for Rare Liver Diseases (ERN RARE‐LIVER), and all centers are expert centers for autoimmune liver diseases. No a priori power calculation was performed; every centre aimed to include as many patients as possible in the given time frame. Data analysis of this study was carried out by a researcher at the University Medical Center Hamburg‐Eppendorf in Germany who was not involved in data assessment.

### 2.2 Participants and Recruitment

Patients with PBC, PSC, AIH as well as with PBC‐AIH or PSC‐AIH variant syndromes were enrolled. Inclusion criteria were (1) a clinical diagnosis, based on the simplified criteria and/or EASL criteria [27, 28], (2) an age of at least 18 years, (3) written informed consent and (4) ability to read and understand the documents and questionnaires, which were handed out in the respective native language. In lack of a generally accepted and validated definition for variant syndromes of cholestatic liver diseases, the diagnosis of variant syndrome was given by the treating physicians and generally based on a diagnosis of PBC/PSC with a disproportionate elevation of transaminase and serum IgG levels, and included liver histology in the majority of cases. Exclusion criteria were (1) a history of liver transplantation or (2) hepatobiliary malignancy. Based on their medical history, physicians of the participating centers assessed whether patients meet inclusion criteria for the study and informed eligible patients about the possibility of participating. Interested patients signed an informed consent form. The study protocol conforms to the ethical guidelines of the 1975 Declaration of Helsinki as reflected in an ethics approval by an independent local ethics committee (2022‐100 929‐BO‐ff).

### 2.3 Data Assessment

Data included clinical records and patient‐reported outcomes (PROs). Clinical data were retrieved from patient records within routine care. PROs were collected with a paper‐pencil survey. The survey was handed to the patients during the routine visit at their outpatient clinic or sent to their home. Patients completed the survey and handed or sent it back to the clinic. Research personnel entered the data in eCRFs using CastorEDC (provided by Ciwit B.V., Amsterdam). Patients completed the survey in their native language. Official translations of the instruments were used if available. In case the respective language was not available, we applied back and forward translations according to a translational protocol. For this study, we analysed only variables relevant for our research question, using mainly PRO.

### 2.4 Variables

#### 2.4.1 Demographical and Clinical Variables

We asked patients about their gender, age, years of formal education, employment status, relationship status, income and comorbidities. The diagnosis and information on whether the patients have cirrhosis were retrieved from patients' medical records. HRQOL was assessed with the Health Survey Short‐Form 12 (SF‐12 version 2) [29], fatigue was assessed with the Modified Fatigue Impact Scale (MFIS) [30], depression severity was assessed with the depression module of the Patient Health Questionnaire‐9 (PHQ‐9) [31], anxiety severity with the Generalised Anxiety Disorder 7‐item scale (GAD‐7) [32, 33], somatic symptom severity with the Somatic Symptom Scale (SSS‐8) [34] and psychological burden by somatic symptoms with the Somatic Symptom Disorder B—Criteria—Scale (SSD‐12) [35]. Detailed information on the scales is in the Supporting Information Section SI.

### 2.5 Data Analysis

We calculated means and standard deviations for continuous data and absolute and relative frequencies for categorical data. For depression severity and anxiety severity, we determined the proportion of patients reaching the cut‐off for clinically relevant symptoms (PHQ‐9/GAD‐7 ≥ 10). Expecting unequal variance between the groups, we exploratively compared means using one‐way Welch‐ANOVA with Games‐Howell post hoc tests. Proportions were compared with Chi‐Square tests including z‐tests. To investigate whether having a variant syndrome contributes to reduced quality of life, we performed a 5‐step hierarchical linear regression analyses with two different subsets of patients: Patients with PBC or PBC‐AIH and patients with PSC or PSC‐AIH. The dependent variable was either physical or mental HRQOL. In the first block of the hierarchical regression models, we added gender and age as control variables. In the second block, we added the diagnosis of a variant syndrome as a binary variable (0 = no variant syndrome, 1 = variant syndrome) to evaluate whether it explains additional variance of HRQOL compared to the cholestatic disease alone (after controlling for gender and age). In a third block, we added cirrhosis (yes/no) to examine whether liver disease stage contributed to differences in HRQOL. In blocks four and five, we included fatigue and depression severity, respectively, as these variables have consistently shown to be associated with HRQOL in prior studies. The step‐wise approach enabled assessment of the incremental contribution of each clinical and psychosocial variable and quantification of changes in explained variance (Δ*R* ^2^) between blocks. By sequentially controlling for confounding variables, the models allowed us to determine the relative importance of individual predictors. Assumptions for linear regression models were checked for all models as follows: Normal distribution of residuals as well as homoscedasticity were examined graphically using normality plots of residuals and scatterplots. To check if residuals were auto‐correlated, we used the Durbin‐Watson‐Test. Multicollinearity was assessed with the Variance Inflation Factor (VIF).

### 2.6 Patient Involvement

Patient representatives and patient advocates were involved in all stages of the research process, providing advice throughout the entire study. They gave comments on the study protocol and shaped the research question and selection of questionnaires. They also advised how to embed and ensure the patient's perspective in this study. In addition, the study was repeatedly discussed within the quality of life working group of the ERN RARE LIVER, of which patient representatives and advocates are an integral part. They will also disseminate the results within their networks on a national and international level. A patient representative/advocate (JAW) is a co‐author of this study.

## 3 Results

### 3.1 Sample Characteristics

**FIGURE 1:**
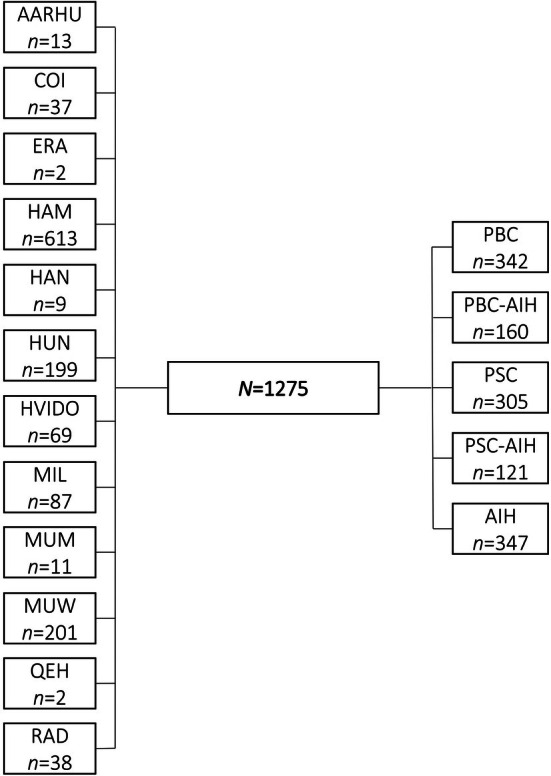
Recruitment details. AARHU, Aarhus University Hospital; AIH, autoimmune hepatitis; COI, Centro Hospitalar e Universitário de Coimbra; ERA, Erasmus University Medical Centre, Rotterdam; HAM, University Medical Center Hamburg‐Eppendorf; HAN, Hannover Medical School; HUN, University of Debrecen; HVIDO, Hvidovre University Hospital Copenhagen; MIL, University of Milano‐Bicocca; MUM, MUMC+, Maastricht; MUW, Medical University of Warsaw; PBC, primary biliary cholangitis; PSC, primary sclerosing cholangitis; QEH, Queen Elizabeth Hospital, University Hospital of Birmingham; RAD, Radboud University Medical Centre.

Across 12 study sites, *N* = 1275 patients were included (PBC: *n* = 342, PBC‐AIH: *n* = 160, PSC: *n* = 305, PSC‐AIH*: n* = 121, AIH: *n* = 347). Recruitment details can be found in Figure 1. The mean age was *M* = 51.2 years (SD = 16.3), 29.9% were male, 70.1% female and no one reported to be non‐binary or have a different gender.

### 3.2 Differences Between the Diagnostic Groups

#### 3.2.1 Demographical and Clinical Characteristics

Table 1 shows an overview of the demographical and clinical characteristics for the different diagnoses and *p*‐values for the statistical comparison of PBC and PBC‐AIH as well as PSC and PSC‐AIH. Statistical comparisons of all groups are shown in the Supporting Information (Section SII; Tables S1 and S2). To summarise, the demographical characteristics of patients with variant syndromes are similar to the primary cholestatic disease, while patients with PBC and PBC‐AIH were older and more often female, and patients with PSC and PSC‐AIH were younger and more often males, with an overall higher education and active employment status. Patients with variant syndromes showed higher rates of cirrhosis than patients with the pure forms of cholestatic liver diseases.

**TABLE 1:** Demographical and clinical characteristics by diagnosis.

Variable	*N*_missing_	PBC	PBC‐AIH	PSC	PSC‐AIH	AIH	*p*	*p*
							PBC vs. PBC‐AIH	PSC vs. PSC‐AIH
***N***	0	342	160	305	121	347		
Age *M* (SD)	3	60.1 (11.1)	58.0 (13.1)	43.8 (14.2)	36.0 (13.5)	51.3 (16.2)	0.389	< **0.001**
Education (years) *M* (SD)	61	12.3 (4.7)	13.6 (5.0)	14.9 (4.7)	14.8 (4.2)	13.1 (4.6)	0.063	0.999
Time since diagnosis (years) *M* (SD)	94	6.4 (6.0)	8.6 (7.2)	7.8 (6.7)	8.0 (7.6)	8.9 (8.2)	0.**015**	0.998
ALT *M* (SD)	15	32.4 (29.7)	58.2 (95.1)	62.6 (74.3)	79.4 (91.7)	50.5 (89.2)	0.**009**	0.388
ALP *M* (SD)	19	137.0 (102.8)	127.4 (96.5)	213.7 (192.5)	205.0 (161.0)	89.5 (59.6)	0.**011**	0.211
IgG *M* (SD)	75	12.9 (6.2)	15.2 (6.6)	13.9 (4.6)	15.9 (5.3)	14.1 (5.9)	0.**004**	0.**004**
Bilirubin (mg/dL) *M* (SD)	296	0.7 (1.1)	0.8 (0.7)	1.0 (1.5)	1.4 (1.6)	1.0 (1.0)	0.859	0.364
Gender *n* (%)	4							
Female		314 (92%)	139 (87%)	131 (43%)	43 (36%)	266 (77%)	0.102	0.171
Employment *n* (%)	44							
Employed		144 (43%)	79 (53%)	219 (75%)	69 (58%)	197 (58%)	0.126	< **0.001**
Retired		134 (40%)	55 (37%)	28 (10%)	8 (7%)	92 (27%)		
Homemaker		25 (8%)	4 (3%)	6 (2%)	4 (3%)	22 (7%)		
On disability		20 (6%)	5 (3%)	7 (2%)	10 (8%)	9 (3%)		
Student		1 (0%)	0 (0%)	21 (7%)	22 (19%)	12 (4%)		
Unemployed		8 (2%)	5 (3%)	13 (4%)	6 (5%)	6 (2%)		
Income *n*(%)	319							
< Median		109 (46%)	47 (42%)	46 (18%)	28 (29%)	84 (33%)	0.439	0.**029**
Relationship *n* (%)	20							
Single		65 (19%)	29 (18%)	83 (28%)	49 (41%)	82 (24%)	0.501	0.**014**
With partner		233 (70%)	116 (74%)	213 (71%)	71 (59%)	240 (70%)		
Widowed		26 (10%)	12 (8%)	5 (2%)	0 (0%)	21 (6%)		
Cirrhosis *n* (%)	86							
Yes		36 (11%)	28 (29%)	28 (11%)	20 (28%)	75 (22%)	0.**008**	< **0.001**
Comorbidities *n* (%)	26							
Yes		197 (59%)	85 (55%)	203 (67%)	79 (66%)	147 (43%)	0.312	0.836
IBD *n* (%)	963							
Yes		NA	NA	158 (60%)	24 (50%)	NA	NA	0.203
Corticosteroid intake *n* (%)	3							
Yes		8 (3%)	53 (19%)	10 (4%)	65 (20%)	147 (54%)	< **0.001**	< **0.001**

*Note:* All variables were assessed at the time of the HRQOL assessment. Bold values indicate statistically significant results (*p* < 0.05).

Abbreviations: AIH, autoimmune hepatitis; PBC, primary biliary cholangitis; PSC, primary sclerosing cholangitis.

#### 3.2.2 Patient‐Reported Outcomes

An overview of HRQOL, fatigue, depression and anxiety severity, somatic symptom severity and psychological burden through somatic symptoms by diagnostic group is displayed in Table 2. *p*‐values of statistical comparisons between PBC, PSC and their respective variant syndrome are shown in Table 2. Statistical comparisons of all groups are in the Supporting Information (Section S2 and Table S3). To summarise, PRO scores were similar in patients with PBC and PBC‐AIH. Both groups had higher fatigue values and lower physical quality of life compared to people with PSC or AIH alone. Patients with a PSC‐AIH variant syndrome showed higher psychopathological and fatigue burden compared to the cholestatic disease alone.

**TABLE 2:** Quality of life, psychopathology and fatigue outcomes by diagnosis.

Variable	*N*_missing_	PBC	PBC‐AIH	PSC	PSC‐AIH	AIH	*p*	*p*
							PBC vs. PBC‐AIH	PSC vs. PSC‐AIH
*N*	0	342	160	305	121	347		
Physical HRQOL (SF‐12)	41	43.6 (11.1)	42.2 (11.5)	49.5 (8.6)	48.1 (9.9)	46.8 (10.5)	0.705	0.641
Mental HRQOL (SF‐12)	41	47.5 (11.0)	45.9 (10.3)	47.4 (10.7)	44.4 (10.8)	47.3 (11.0)	0.562	0.069
Physical functioning (SF‐12)	15	44.6 (11.3)	44.6 (11.3)	51.7 (8.4)	50.8 (8.7)	48.4 (10.7)	1.000	0.885
Role physical (SF‐12)	32	43.7 (11.2)	42.1 (11.1)	48.2 (9.9)	44.4 (10.6)	46.0 (11.3)	0.541	0.**007**
Bodily pain (SF‐12)	25	46.1 (12.1)	43.7 (13.1)	50.0 (10.2)	48.4 (11.6)	47.1 (11.6)	0.283	0.656
General health (SF‐12)	30	40.2 (10.6)	38.4 (11.3)	43.4 (10.3)	42.2 (10.7)	42.9 (10.4)	0.471	0.830
Vitality (SF‐12)	48	47.8 (10.8)	46.5 (11.2)	49.6 (9.7)	47.8 (10.5)	49.0 (10.5)	0.730	0.494
Social functional (SF‐12)	18	46.6 (11.4)	43.5 (12.3)	48.2 (10.7)	46.0 (10.8)	46.6 (11.3)	0.062	0.338
Role emotional (SF‐12)	30	44.5 (12.7)	43.2 (11.1)	47.0 (11.5)	44.3 (11.6)	45.8 (12.4)	0.825	0.199
Mental health (SF‐12)	16	47.6 (10.3)	46.4 (10.4)	48.6 (10.0)	45.3 (9.0)	48.3 (10.5)	0.750	0.**013**
Fatigue (MFIS)	151	23.2 (20.4)	26.5 (17.7)	17.0 (17.8)	22.5 (18.4)	18.8 (16.1)	0.468	0.**029**
Depression severity (PHQ‐9)	46	5.8 (5.3)	6.2 (4.8)	5.0 (4.7)	6.4 (4.6)	5.0 (4.8)	0.907	0.**035**
Anxiety severity (GAD‐7)	41	5.1 (4.5)	5.0 (4.7)	4.3 (4.1)	5.9 (4.6)	4.3 (4.3)	1.000	0.**009**
Somatic symptom severity (SSS8)	18	9.1 (6.5)	9.4 (5.5)	6.5 (5.3)	7.7 (5.6)	7.6 (5.8)	0.982	0.274
Psychological burden through symptoms (SSD‐12)	98	12.5 (10.7)	13.4 (9.6)	11.9 (10.1)	13.2 (9.4)	11.3 (9.9)	0.911	0.696

*Note:* All variables were assessed at the time of the HRQOL assessment. Bold values indicate statistically significant results (*p* < 0.05).

Abbreviations: AIH, autoimmune hepatitis; PBC, primary biliary cholangitis; PSC, primary sclerosing cholangitis.

#### 3.2.3 Psychopathology Screening

**FIGURE 2:**
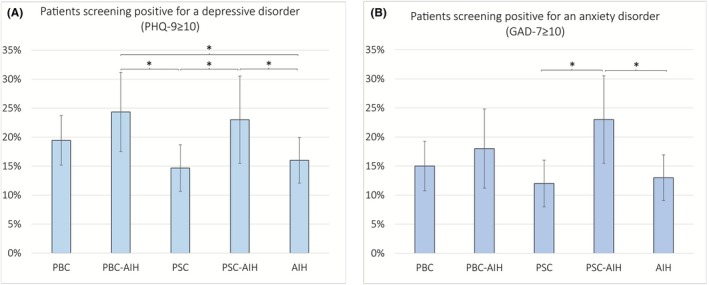
Rates of patients with increased psychopathology by diagnosis. Error bars show 95% confidence intervals for proportions (normal approximation to the binomial calculation); *indicate significant group differences at the 0.05 level. The corresponding table can be found in the Supporting Information. AIH, autoimmune hepatitis; PBC, primary biliary cholangitis; PSC, primary sclerosing cholangitis.

Figure 2 shows the proportion of patients who screened positive for a depressive disorder (PHQ‐9 ≥ 10) and an anxiety disorder (GAD‐7 ≥ 10) according to the different diagnoses (see Supporting Information, Section SIII and Table S4 for respective data). Overall, patients with variant syndromes showed higher depression and anxiety rates. For depression severity, we repeated the group comparison including the intake of corticosteroids (yes/no) as a covariate. Corticosteroid intake was not associated with depression severity (*F* (1) = 1.34, *p* = 0.247), and the observed group differences remained significant.

### 3.3 Regression Analyses

**FIGURE 3:**
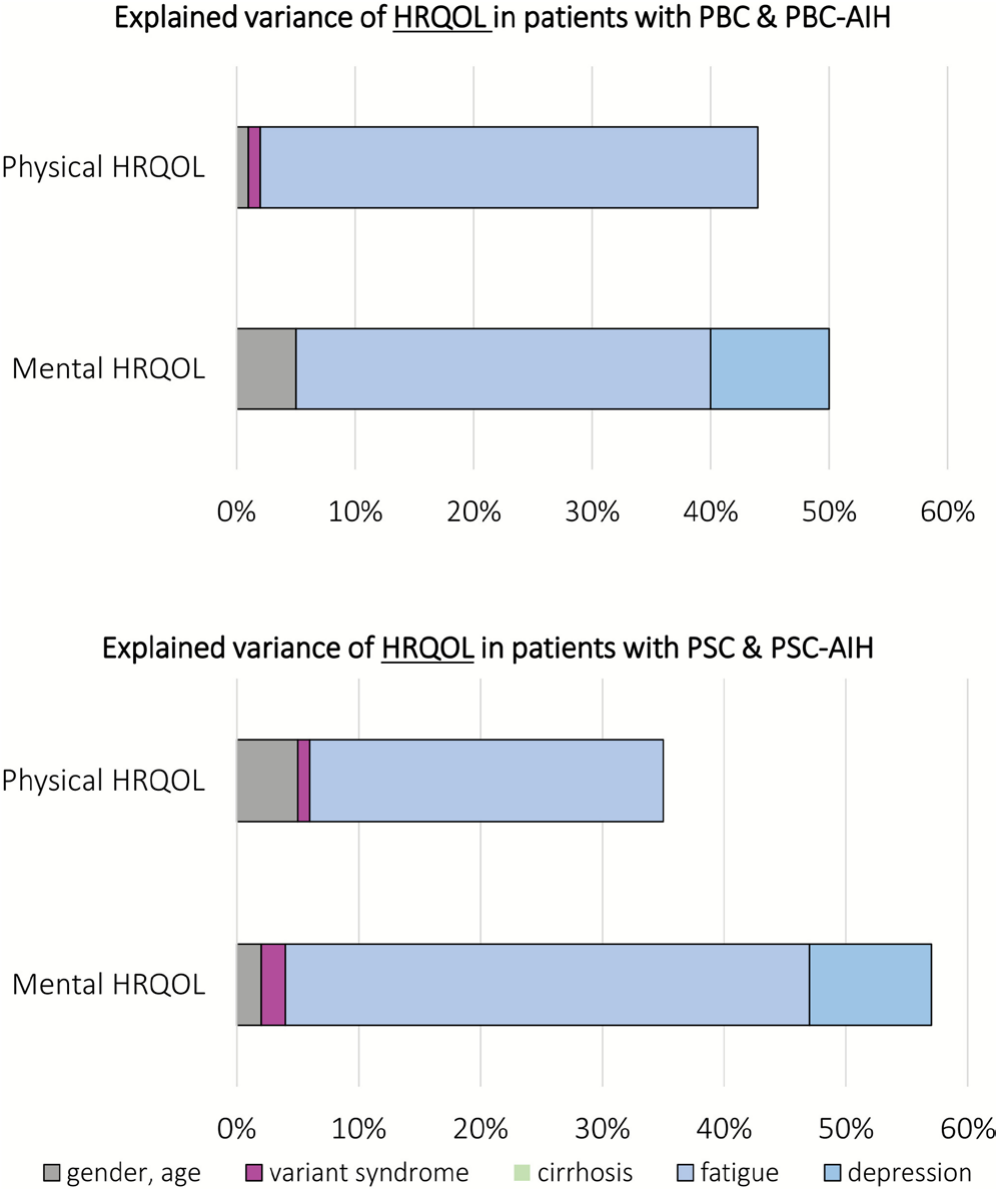
Degree of explained variation of HRQOL for the different blocks of the regression model. AIH, autoimmune hepatitis; PBC, primary biliary cholangitis; PSC, primary sclerosing cholangitis.

We performed hierarchical regression analyses to evaluate whether having a variant syndrome significantly contributes to reduced HRQOL compared to having the cholestatic liver disease alone. For all models, the assumptions for regression analysis were met. The full tables for all models can be found in the Supporting Information (Section SIV; Tables S5–S8). A visualisation of the degree of explained variation for each model can be found in Figure 3.

### 3.4 HRQOL in Patients With PBC and PBC‐AIH

#### 3.4.1 Physical HRQOL

In patients with PBC and PBC‐AIH, the first block including gender and age did not significantly explain variation of physical HRQOL (Δ*R* ^2^ = 0.014, *p* = 0.074). Adding the diagnosis of a variant syndrome did not improve the model (Δ*R* ^2^ = 0.008, *p* = 0.081). Adding cirrhosis did not improve the model (Δ*R* ^2^ = 0.002, *p* = 0.358), while adding fatigue led to a significant increase in explained variance of 42% (Δ*R* ^2^ = 0.418, *p* < 0.001). Depression severity did not improve the model (Δ*R* ^2^ = 0.002, *p* = 0.223). The final model explained 44% of the variance in physical HRQOL and significant predictors were age (*B* = −0.201, *p* < 0.001) and fatigue (*B* = −0.417, *p* < 0.001).

#### 3.4.2 Mental HRQOL

The first block with gender and age significantly explained variance of mental HRQOL (Δ*R* ^2^ = 0.052, *p* < 0.001). Having a PBC‐AIH variant syndromes did not significantly contribute to explained variation in HRQOL (Δ*R* ^2^ = 0.001, *p* = 0.609) with PBC being the reference category. The third block with cirrhosis did not improve the model (Δ*R* ^2^ = 0.000, *p* = 0.809), while fatigue (Δ*R* ^2^ = 0.354, *p* < 0.001) and depression severity (Δ*R* ^2^ = 0.095, *p* < 0.001) did. In the final model, age (*B* = 0.087, *p* = 0.015), depression severity (*B* = −1.120, *p* < 0.001) and fatigue (*B* = −0.108, *p* = 0.001) were significant predictors of mental HRQOL. The final model explained 50% of the variance of mental HRQOL.

To summarise, being affected by a PBC‐AIH variant syndrome did not contribute to reduced physical or mental quality of life compared to PBC alone. Higher age and fatigue were associated with reduced physical HRQOL. Lower age, fatigue and higher depression severity were associated with reduced mental HRQOL.

### 3.5 HRQOL in Patients With PSC and PSC‐AIH

#### 3.5.1 Physical HRQOL

In patients with PSC and PSC‐AIH, gender and age significantly explained variation of HRQOL (Δ*R* ^2^ = 0.054, *p* < 0.001). Adding the diagnosis of a variant syndrome significantly improved the model (Δ*R* ^2^ = 0.012, *p* = 0.041). Cirrhosis did not improve the model (Δ*R* ^2^ = 0.002, *p* = 0.361). Fatigue led to a significant increase of explained variance (Δ*R* ^2^ = 0.288, *p* < 0.001). Depression severity did not further improve the model (Δ*R* ^2^ = 0.000, *p* = 0.679). The final model explained 36% of physical HRQOL and significant predictors were age (*B* = −0.107, *p* < 0.001) and fatigue (*B* = −0.289, *p* < 0.001), while the diagnosis of a variant syndrome was no longer associated with HRQOL.

#### 3.5.2 Mental HRQOL

In patients with PSC and PSC‐AIH, the first block significantly explained the variance of mental HRQOL (Δ*R* ^2^ = 0.024, *p* = 0.015). The second block with the diagnosis of a variant syndrome further contributed to the explained variation of mental HRQOL (Δ*R* ^2^ = 0.016, *p* = 0.020). Adding cirrhosis with the third block did not improve the model (Δ*R* ^2^ = 0.002, *p* = 0.392). Both fatigue (Δ*R* ^2^ = 0.425, *p* < 0.001) and depression severity (Δ*R* ^2^ = 0.103, *p* < 0.001) significantly contributed to the explained variance. Significant predictors in the final model were depression severity (*B* = −1.248, *p* < 0.001) and fatigue (*B* = −0.135, *p* < 0.001), while the variant syndrome was no longer a significant predictor. The final model explained 57% of the variance.

To summarise, being affected by a PSC‐AIH variant syndrome significantly contributed to both reduced physical and mental HRQOL compared to PSC alone, when controlling for gender and age. This effect disappeared when adding cirrhosis, fatigue and depression severity to the model. Higher age and more fatigue were associated with reduced physical HRQOL, higher fatigue values and higher depression severity with reduced mental HRQOL.

Cirrhosis was not an independent predictor of HRQOL in any of the models. Fatigue had the strongest impact on both physical and mental HRQOL and in both patients with PBC/PBC‐AIH and PSC/PSC‐AIH.

### 3.6 HRQOL in Patients With AIH and Both Variant Syndromes

We additionally investigated in an exploratory manner whether being affected by a variant syndrome contributes to reduced quality of life compared to AIH. To summarise, compared to AIH alone, a PBC‐AIH variant syndrome significantly contributed to reduced physical, but not to mental HRQOL. Having a PSC‐AIH variant syndrome contributed to both reduced physical and mental HRQOL compared to AIH alone. Higher age, fatigue and cirrhosis were associated with physical HRQOL. Lower age, fatigue and depression severity were associated with mental HRQOL. The detailed results can be found in the Supporting Information (Section SV, Tables S9–S12).

## 4 Discussion

This cross‐sectional study aimed to gain insights into HRQOL in patients with variant syndromes of autoimmune liver diseases across nine European countries. Patients with variant syndromes showed high physical and mental burden, particularly those affected by PSC‐AIH. Fatigue was identified as the most prominent factor associated with mental and physical HRQOL.

Having PSC‐AIH contributed to reduced physical and mental HRQOL compared to PSC (and AIH) alone, when controlling for gender and age. This supports our assumption that the higher clinical complexity of variant syndromes may go along with higher physical burden for patients, which is in line with previous studies indicating a worse prognosis in these conditions [24, 25, 26]. Environmental factors such as disease‐associated microbiota composition and biological factors such as age might be involved [36]. Systemic inflammation has been proposed to disrupt neurotransmitter balance and cause neuroinflammation, potentially explaining the link to fatigue [14] and depression [37] and effects of intestinal microbiota on cognitive function, HRQOL and fatigue have been reported recently [38, 39, 40]. Patients with PSC‐AIH are a subgroup of patients facing complex and often invasive therapies and a high risk of disease progression, potentially explaining why we found a particularly high physical and mental burden in this group. Although patients with PSC‐AIH showed lower HRQOL scores when only age and sex were controlled for, this effect was no longer present after cirrhosis, fatigue and depression severity were added to the model, with fatigue having the strongest impact. This indicates that the initially observed difference is fully accounted for by the higher symptom burden in PSC‐AIH rather than by the variant syndrome itself.

In patients with PBC, the AIH overlap syndrome showed a marginal association with physical HRQOL when only age and sex were controlled for, but this effect was not statistically significant. It suggests that additional AIH in a cholestatic liver disease alone does not lead to more severely impaired HRQOL and that more variables need to be considered to understand what impacts patients' well‐being. Being affected by the variant syndrome may not substantially worsen HRQOL beyond what is already experienced in PBC alone. This is supported by a variety of similar demographic and clinical characteristics we found in these two groups and the overall good prognosis if treated adequately. Overall, the phenotypes of variant syndromes generally seem to be closer to PBC and PSC than to AIH, supporting the assumption that these syndromes are primarily cholestatic liver diseases with additional AIH.

Age, fatigue and depression severity were confirmed as relevant predictors of HRQOL in this study. This is in line with previous research, as summarised in a recent review [10]. Higher age was associated with lower physical but higher mental quality of life, although the coefficient for the latter association was rather small. Lower physical wellbeing in older individuals could be explained by a generally higher physical symptom burden and higher rates of comorbidities. That mental HRQOL was higher in older patients could be explained by psychological mechanisms such as adjusting to one's condition [41]. Disease acceptance, as a possible outcome of disease adjustment, has been shown to be associated with quality of life in chronically ill patients [42, 43]. Being of older age and therefore living longer with a chronic condition may lead to a better disease adjustment and therefore higher mental well‐being. Although HRQOL was consistently lower in our cohort than in the general population, the age‐related decline in physical and the modest increase in mental HRQOL closely paralleled normative trends, suggesting that the association between age and HRQOL reflects normative aging rather than disease‐specific acceleration. Fatigue is a very common complaint causing burden for many patients with chronic liver diseases [44]. In our study, fatigue consistently emerged as a significant predictor, confirming its substantial impact on both physical and mental aspects of HRQOL across various autoimmune liver diseases, including PSC [16]. The same applies to depression severity. Depression has been found to be one of the strongest predictors of quality of life in autoimmune liver diseases [9, 17, 20], other chronic conditions [45] and in population‐based studies [46]. Our study confirms the impact that depressive symptoms have on patients' mental wellbeing. In the models that included AIH (Supporting Information), cirrhosis was another relevant predictor of reduced physical HRQOL. This is also in line with previous research [17, 18].

The rates of patients screening positive for a depressive and an anxiety disorder ranged from 16% to 24% and 12% to 23%, respectively, with patients affected by a PSC‐AIH variant syndrome displaying the highest symptom burden. The proportions in all groups were higher than in the general population. In a large cohort study from the German population using the same screening tools, the authors found increased depression rates in about 8% of the participants [47] and about 6% screened positive for an anxiety disorder [48]. Our results are in line with evidence from previous studies on psychopathology across different rare chronic conditions [49] and in autoimmune liver diseases [9, 21]. However, to our knowledge, psychopathology has not been compared between different autoimmune liver diseases and their variant syndromes. Our data indicate high psychological distress in patients with variant syndromes, particularly in PSC‐AIH. Comorbid depression is linked to medication non‐adherence [50, 51]. Particularly in patients with AIH, adhering to long‐term immunosuppression is crucial to induce biochemical and histological remission, prevent relapses and improve the clinical course. Therefore, detecting and treating comorbid depression early is crucial. Incorporating routine screening for mental diseases into clinical assessments can help identify patients who may benefit from additional support and psychological interventions. Interestingly, corticosteroids were not associated with depression severity, while previous research showed that corticosteroid use was associated with depressive symptoms [21].

Our study has several strengths and limitations. Due to the collaboration of 12 different centers across Europe, we were able to recruit a large number of patients. To date, this is the largest sample of patients with variant syndromes of autoimmune liver diseases. Standardised diagnostic criteria and recruitment processes ensured comparability between the study sites. However, this study has some limitations. Firstly, the study design is cross‐sectional, which limits the ability to infer causality. Our study discovered associations between variables but cannot establish temporal relationships or causative effects. For instance, it remains unclear whether depressive symptoms lead to a reduced quality of life or if they are rather a consequence of reduced physical well‐being. Secondly, the study relied on PROs, which may be influenced by recall bias or social desirability bias. Thirdly, while we controlled for gender and age, other potential confounders such as comorbidities or medication use have not been accounted for. Although comorbidities were associated with HRQOL in previous studies [19], we decided against including them in our analysis as they were assessed as a binary item solely based on self‐report. A particularly frequent and potentially relevant comorbidity in patients with PSC is IBD. However, as we wanted to ensure comparability between the different models and therefore include the same variables for all subgroup analyses, we did not include this aspect either. In addition, IBD in PSC is of mild activity in most patients. Forth, medication use was not considered. Previous research showed that corticosteroid use was associated with depressive symptoms [21] and may therefore be a confounder, which was not considered in our analysis. Diagnostic uncertainty may be another variable affecting mental HRQOL, especially in variant syndromes where classification is less straightforward and patients may face greater ambiguity. Although not captured in our study, this psychosocial dimension may partly contribute to elevated anxiety or reduced mental well‐being and warrants consideration in future research. The predictors we included explained up to half of the variation of HRQOL. While this is comparable to other studies on determinants of HRQOL, we do not claim to provide a comprehensive picture of the factors influencing quality of life in patients with autoimmune liver diseases. Lastly, the diagnosis of variant syndromes could not be corroborated with standardised serological or histological markers because these data were not consistently available, and clear diagnostic criteria remain lacking.

## 5 Conclusion

This cross‐sectional multicenter study provides the first large‐scale assessment of physical and mental well‐being in patients with variant syndromes of autoimmune liver diseases. Patients with variant syndromes showed substantial physical and mental health impairments. The PSC‐AIH variant syndrome was associated with reduced HRQOL compared to the cholestatic disease alone, but this effect is likely explained by a higher symptom burden, rather than the variant syndrome itself. In PBC, the additional diagnosis of AIH showed no significant contribution to HRQOL, underlining the dominant influence the disease itself exerts. Fatigue was the strongest predictor of HRQOL in all groups. Together, these findings highlight that symptom burden—particularly fatigue and depression—accounts for a substantial proportion of HRQOL impairment, and that patients with variant syndromes represent a vulnerable subgroup who may benefit from targeted supportive interventions.

## Author Contributions

N.U.: data analysis, writing – original draft preparation, R.J.A.L.M.S.: study coordinator, data acquisition, writing – critical revision of the manuscript, T.J.G.G. and P.M. conceived the study, C.S. and T.J.G.G. supervised this study. All remaining authors: data acquisition, critical revision of the manuscript. All the authors have approved the final version of the manuscript.

## Funding

This study was supported by the Medical University of Warsaw, Warsaw, Poland (SF‐12 licence Nno. QM052505) and the FIM Foundation, Czestochowa, Poland (data collection phase of this study). CS and MM were supported by the Helmut and Hannelore Greve Foundation, and the YAEL Foundation. BE was supported by the PRACTIS—Clinician Scientist Program of Hannover Medical School, funded by the German Research Foundation (DFG, ME 3696/3). AG and PI were supported by Italian MUR Dipartimenti di Eccellenza 2023–2027 (l. 232/2016, art. 1, commi 314–337), Italian MUR PNRR PE06 “HEAL ITALIA–Health Extended ALliance for Innovative Therapies, Advanced Lab‐research and Integrated Approaches of Precision Medicine”—Spoke 4—Precision Diagnostics, Italian MUR PRIN 2022 PNRR P2022H7JYZ “Non‐invasive biological and molecular characterization of autoimmune liver diseases and variant syndromes”. Y.H.O. are supported by Wellcome Trust Discovery award (reference: 326757/Z/25/Z), Sir Jules Thorn Charitable Trust (reference: 18JTA), Whitney wood Scholarship awarded by the Royal College of Physicians and University Hospital Birmingham Charity and NIHR Birmingham BRC. HG received research grants (Abbvie, Intercept, ARLA Food for Health, ADS AIPHIA Development Services AG) and consulting fees (NOVO, Pfizer, Astrazeneca), was lecturer at AstraZeneca, EISAI and in a Data Monitoring Committee: CAMURUS AB. The European Reference Network for Hepatological Diseases, ERN RARE‐LIVER, is supported by the European Commission.

## Ethics Statement

The study protocol conforms to the ethical guidelines of the 1975 Declaration of Helsinki as reflected in an ethics approval by an independent local ethics committee (2022‐100 929‐BO‐ff). All patients provided written informed consent.

## Conflicts of Interest

The authors declare no conflicts of interest.

## Supporting information


**Appendix S1:** liv70526‐sup‐0001‐AppendixS1.docx.

## Data Availability

The data that support the findings of this study are available from the corresponding author upon reasonable request.
