# Supplementary material for: Quality of Life in Patients With Variant Syndromes of Autoimmune Liver Diseases—A Cross‐Sectional Multicentre Study
Source: Liver Int. 2026 Feb 2;46(3):e70526. doi: 10.1111/liv.70526 (PMC12862555; doi:10.1111/liv.70526)
Supplement: Supplementary file 1 — Appendix S1: liv70526‐sup‐0001‐AppendixS1.docx. [file LIV-46-0-s001.docx]

**SUPPLEMENTARY MATERIAL**

1. **DETAILED INFORMATION ON THE PATIENT-REPORTED OUTCOME SCALES**

*Health-related quality of life.* To measure HRQOL, the Health Survey Short-Form 12 (SF-12 version 2) was used. The SF-12 consists of twelve questions addressing eight domains: physical function, role physical, bodily pain, general health, mental health, role emotional, social function and vitality. Further, the first four domains form the physical component score (PCS), while the remaining four form the mental component score (MCS). Scales range for the PCS and MCS were standardized for the 2009 U.S. general population with a mean (*M*) of 50 and a standard deviation (*SD*) of 10. The SF-12 is a valid and reliable instrument^1^ that has been validated across countries^2^ and has been applied in the context of different chronic conditions, including patients with autoimmune liver diseases^3-5^.

*Fatigue.* We assessed fatigue with the Modified Fatigue Impact Scale (MFIS), a 21-items version of the longer Fatigue Impact Scale. It assesses the impact of fatigue on physical, cognitive and psychosocial levels and offers a total score. It was initially developed for and validated with patients with multiple sclerosis^6^, but has been applied in a variety of conditions, including patients with PBC^7^. It has been evaluated across different European countries and the instrument demonstrated reliability and the total score showed very good internal consistency^8^.

*Depression severity*. Depression severity was assessed with the depression module of the Patient Health Questionnaire-9 (PHQ-9)^9^. It is a reliable instrument whose validity has been demonstrated for various populations ^10-12^. With nine items, depression symptoms are measured on a scale from 0 (not bothered at all) to 3 (bothered almost every day). As a single cut-off value indicating a depressive disorder, a sum-score of **≥**10 is recommended ^13^.

*Anxiety severity.* We measured anxiety with the Generalized Anxiety Disorder 7-item scale (GAD-7)^14,15^, a screening instrument to determine anxiety severity and identity of different anxiety disorders ^16^. A sum-score value of ≥10 being the recommended cut-off for a positive anxiety disorder screening ^14^. The scale has a very good internal consistency ^15^, good test-retest reliability and showed criterion, construct, factorial, and procedural validity ^14^.

*Somatic symptom severity.* The Somatic Symptom Scale (SSS-8) is a short version of the PHQ-15, a widely used, valid and reliable self-report measure of somatic symptom severity^17,18^. The scale has eight items to measure somatic symptom severity on a scale from 0 (“not bothered at all”) to 2 (“bothered a lot”) for each symptom. Sum score values of ≥5, ≥10, ≥15 indicate mild, moderate, and severe somatic symptom burden, respectively. The instrument’s validity and reliability has been demonstrated^19^.

*Psychological burden through somatic symptoms.* The Somatic Symptom Disorder B - Criteria – Scale (SSD-12) was used to measure patients' perceptions of their symptom-related thoughts, feelings, and behaviors. It has been developed to assess the B-criterion of the DSM-5 Somatic Symptom Disorder. The 12-item instrument has an excellent internal consistency and is reliable and valid in measuring psychological characteristics related to the experience of somatic symptoms^20,21^.

1. **COMPARISONS OF ALL DIAGNOSTIC GROUPS (SUPPLEMENTING TABLE 1 and 2)**

**Table S1. Statistical comparisons of diagnostic groups for continuous demographic and clinical variables in Table 1.**

| **Dependent variable** | **Diagnostic group** | **Diagnostic group** | **Mean difference** | **Standard error** | **Sig.** | **95% Confidence interval** | |
| --- | --- | --- | --- | --- | --- | --- | --- |
|  |  |  |  |  |  | **Lower** | **Upper** |
| **Age** | PBC | PSC | 16,33524* | 1,00686 | <,001 | 13,5799 | 19,0906 |
|  |  | PBC-AIH | 2,17627 | 1,23475 | ,398 | -1,2161 | 5,5686 |
|  |  | PSC-AIH | 24,16502* | 1,35920 | <,001 | 20,4195 | 27,9105 |
|  |  | AIH | 8,86395* | 1,09541 | <,001 | 5,8666 | 11,8613 |
|  | PSC | PBC-AIH | -14,15897* | 1,35401 | <,001 | -17,8729 | -10,4450 |
|  |  | PSC-AIH | 7,82978* | 1,46838 | <,001 | 3,7926 | 11,8669 |
|  |  | AIH | -7,47129* | 1,22828 | <,001 | -10,8312 | -4,1114 |
|  | PBC-AIH | PSC-AIH | 21,98875* | 1,63309 | <,001 | 17,5026 | 26,4749 |
|  |  | AIH | 6,68768* | 1,42109 | <,001 | 2,7924 | 10,5829 |
|  | PSC-AIH | AIH | -15,30107* | 1,53046 | <,001 | -19,5047 | -11,0974 |
| **ALT** | PBC | PSC | -30,255* | 4,568 | <,001 | -42,78 | -17,73 |
|  |  | PBC-AIH | -25,798* | 7,737 | ,009 | -47,13 | -4,47 |
|  |  | PSC-AIH | -46,993* | 8,529 | <,001 | -70,59 | -23,39 |
|  |  | AIH | -18,144* | 5,068 | ,004 | -32,03 | -4,26 |
|  | PSC | PBC-AIH | 4,457 | 8,687 | ,986 | -19,41 | 28,32 |
|  |  | PSC-AIH | -16,738 | 9,399 | ,388 | -42,63 | 9,15 |
|  |  | AIH | 12,111 | 6,424 | ,326 | -5,46 | 29,68 |
|  | PBC-AIH | PSC-AIH | -21,195 | 11,284 | ,332 | -52,19 | 9,80 |
|  |  | AIH | 7,654 | 8,959 | ,913 | -16,94 | 32,25 |
|  | PSC-AIH | AIH | 28,850* | 9,651 | ,026 | 2,29 | 55,41 |
| **ALP** | PBC | PSC | -76,6805* | 12,3868 | <,001 | -110,606 | -42,754 |
|  |  | PBC-AIH | 9,5927 | 9,4937 | ,850 | -16,450 | 35,635 |
|  |  | PSC-AIH | -68,0063* | 15,7774 | <,001 | -111,561 | -24,452 |
|  |  | AIH | 47,5134* | 6,4586 | <,001 | 29,836 | 65,190 |
|  | PSC | PBC-AIH | 86,2731* | 13,4603 | <,001 | 49,410 | 123,136 |
|  |  | PSC-AIH | 8,6742 | 18,4384 | ,990 | -41,982 | 59,330 |
|  |  | AIH | 124,1939* | 11,5222 | <,001 | 92,601 | 155,787 |
|  | PBC-AIH | PSC-AIH | -77,5990* | 16,6335 | <,001 | -123,433 | -31,765 |
|  |  | AIH | 37,9208* | 8,3342 | <,001 | 14,993 | 60,849 |
|  | PSC-AIH | AIH | 115,5198* | 15,1081 | <,001 | 73,722 | 157,317 |
| **IgG** | PBC | PSC | -,9862 | ,4445 | ,174 | -2,203 | ,230 |
|  |  | PBC-AIH | -2,2589* | ,6371 | ,004 | -4,008 | -,510 |
|  |  | PSC-AIH | -3,0010* | ,5982 | <,001 | -4,645 | -1,357 |
|  |  | AIH | -1,2108 | ,4770 | ,084 | -2,516 | ,094 |
|  | PSC | PBC-AIH | -1,2726 | ,6001 | ,215 | -2,922 | ,377 |
|  |  | PSC-AIH | -2,0147* | ,5586 | ,004 | -3,553 | -,477 |
|  |  | AIH | -,2246 | ,4263 | ,985 | -1,391 | ,942 |
|  | PBC-AIH | PSC-AIH | -,7421 | ,7214 | ,842 | -2,723 | 1,239 |
|  |  | AIH | 1,0481 | ,6245 | ,449 | -,667 | 2,763 |
|  | PSC-AIH | AIH | 1,7902* | ,5848 | ,021 | ,182 | 3,398 |
| **Bilirubin (mg/dl)** | PBC | PSC | -,34000* | ,11559 | ,028 | -,6566 | -,0234 |
|  |  | PBC-AIH | -,08990 | ,09075 | ,859 | -,3387 | ,1589 |
|  |  | PSC-AIH | -,68729* | ,17853 | ,002 | -1,1816 | -,1930 |
|  |  | AIH | -,26588* | ,09082 | ,029 | -,5145 | -,0173 |
|  | PSC | PBC-AIH | ,25010 | ,11239 | ,173 | -,0580 | ,5582 |
|  |  | PSC-AIH | -,34730 | ,19044 | ,364 | -,8729 | ,1783 |
|  |  | AIH | ,07412 | ,11245 | ,965 | -,2339 | ,3822 |
|  | PBC-AIH | PSC-AIH | -,59740* | ,17647 | ,008 | -1,0863 | -,1084 |
|  |  | AIH | -,17598 | ,08670 | ,254 | -,4138 | ,0619 |
|  | PSC-AIH | AIH | ,42142 | ,17651 | ,126 | -,0676 | ,9104 |
| **Time since diagnosis (yrs)** | PBC | PSC | -1,417 | ,531 | ,060 | -2,87 | ,04 |
|  |  | PBC-AIH | -2,205* | ,695 | ,015 | -4,12 | -,29 |
|  |  | PSC-AIH | -1,666 | ,812 | ,247 | -3,91 | ,58 |
|  |  | AIH | -2,569* | ,553 | <,001 | -4,08 | -1,06 |
|  | PSC | PBC-AIH | -,788 | ,737 | ,822 | -2,81 | 1,24 |
|  |  | PSC-AIH | -,249 | ,848 | ,998 | -2,59 | 2,09 |
|  |  | AIH | -1,152 | ,605 | ,316 | -2,81 | ,50 |
|  | PBC-AIH | PSC-AIH | ,539 | ,959 | ,980 | -2,10 | 3,18 |
|  |  | AIH | -,364 | ,753 | ,989 | -2,43 | 1,70 |
|  | PSC-AIH | AIH | -,903 | ,862 | ,833 | -3,28 | 1,47 |
| **Education (yrs)** | PBC | PSC | -2,5994* | ,3764 | <,001 | -3,629 | -1,570 |
|  |  | PBC-AIH | -1,2984 | ,4887 | ,063 | -2,641 | ,044 |
|  |  | PSC-AIH | -2,4922* | ,4646 | <,001 | -3,770 | -1,215 |
|  |  | AIH | -,7509 | ,3626 | ,234 | -1,743 | ,241 |
|  | PSC | PBC-AIH | 1,3010 | ,4949 | ,068 | -,058 | 2,660 |
|  |  | PSC-AIH | ,1072 | ,4712 | ,999 | -1,188 | 1,402 |
|  |  | AIH | 1,8485* | ,3710 | <,001 | ,833 | 2,864 |
|  | PBC-AIH | PSC-AIH | -1,1938 | ,5649 | ,218 | -2,746 | ,358 |
|  |  | AIH | ,5475 | ,4846 | ,791 | -,784 | 1,879 |
|  | PSC-AIH | AIH | 1,7413* | ,4602 | ,002 | ,475 | 3,007 |
| Notes. *Indicates significant mean difference (p<0.05). | | | | | | | |

**Table S2. Statistical comparisons of diagnostic groups for categorical demographic and clinical variables in Table 1.**

|  | | | **PBC** | **PSC** | **AIH-PBC** | **AIH-PSC** | **AIH** | ***p*** |
| --- | --- | --- | --- | --- | --- | --- | --- | --- |
| **Gender** | Male | N | 27_a_ | 173_b_ | 20_a_ | 77_b_ | 81_c_ | <.001 |
|  |  | % | 7,9% | 56,9% | 12,6% | 64,2% | 23,3% |  |
|  | Female | N | 314_a_ | 131_b_ | 139_a_ | 43_b_ | 266_c_ |  |
|  |  | % | 92,1% | 43,1% | 87,4% | 35,8% | 76,7% |  |
| Notes. Subscript letters indicate if diagnostic groups differ from each other on the .05 level. The same letter means there is no statistical difference, a different letter means the groups differ from each other. | | | | | | | | |

|  | | | **PBC** | **PSC** | **AIH-PBC** | **AIH-PSC** | **AIH** | ***p*** |
| --- | --- | --- | --- | --- | --- | --- | --- | --- |
| **Work situation** | Employed | N | 144_a_ | 219_b_ | 79_c_ | 69_c_ | 197_c_ | <.001 |
|  |  | % | 43,4% | 74,5% | 53,4% | 58,0% | 58,3% |  |
|  | Unemployed | N | 8_a_ | 13_a_ | 5_a_ | 6_a_ | 6_a_ |  |
|  |  | % | 2,4% | 4,4% | 3,4% | 5,0% | 1,8% |  |
|  | Homemaker | N | 25_a_ | 6_b_ | 4_b, c_ | 4_a, b, c_ | 22_a, c_ |  |
|  |  | % | 7,5% | 2,0% | 2,7% | 3,4% | 6,5% |  |
|  | Retired | N | 134_a_ | 28_b_ | 55_a_ | 8_b_ | 92_c_ |  |
|  |  | % | 40,4% | 9,5% | 37,2% | 6,7% | 27,2% |  |
|  | On disability | N | 20_a_ | 7_b_ | 5_a, b_ | 10_a_ | 9_b_ |  |
|  |  | % | 6,0% | 2,4% | 3,4% | 8,4% | 2,7% |  |
|  | Student | N | 1_a_ | 21_b_ | 0_a_ | 22_c_ | 12_d_ |  |
|  |  | % | 0,3% | 7,1% | 0,0% | 18,5% | 3,6% |  |
| Notes. Subscript letters indicate if diagnostic groups differ from each other on the .05 level. The same letter means there is no statistical difference, a different letter means the groups differ from each other. | | | | | | | | |

|  | | | **PBC** | **PSC** | **AIH-PBC** | **AIH-PSC** | **AIH** | ***p*** |
| --- | --- | --- | --- | --- | --- | --- | --- | --- |
| **Income** | < median | N | 109_a_ | 46_b_ | 47_a, c_ | 28_d_ | 84_c, d_ | <.001 |
|  |  | % | 46,0% | 18,0% | 41,6% | 28,6% | 33,2% |  |
|  | >median | N | 128_a_ | 209_b_ | 66_a, c_ | 70_d_ | 169_c, d_ |  |
|  |  | % | 54,0% | 82,0% | 58,4% | 71,4% | 66,8% |  |
| Notes. Subscript letters indicate if diagnostic groups differ from each other on the .05 level. The same letter means there is no statistical difference, a different letter means the groups differ from each other. | | | | | | | | |

|  | | | **PBC** | **PSC** | **AIH-PBC** | **AIH-PSC** | **AIH** | ***p*** |
| --- | --- | --- | --- | --- | --- | --- | --- | --- |
| **Relationship status** | Single | N | 65_a_ | 83_b_ | 29_a_ | 49_c_ | 82_a, b_ | <.001 |
|  |  | % | 19,5% | 27,6% | 18,5% | 40,8% | 23,9% |  |
|  | Partner | N | 233_a_ | 213_a_ | 116_a_ | 71_b_ | 240_a_ |  |
|  |  | % | 69,8% | 70,8% | 73,9% | 59,2% | 70,0% |  |
|  | Widowed | N | 36_a_ | 5_b_ | 12_a, c_ | 0_b_ | 21_c_ |  |
|  |  | % | 10,8% | 1,7% | 7,6% | 0,0% | 6,1% |  |
| Notes. Subscript letters indicate if diagnostic groups differ from each other on the .05 level. The same letter means there is no statistical difference, a different letter means the groups differ from each other. | | | | | | | | |

|  | | | **PBC** | **PSC** | **AIH-PBC** | **AIH-PSC** | **AIH** | ***p*** |
| --- | --- | --- | --- | --- | --- | --- | --- | --- |
| **Cirrhosis** | No | N | 292_a_ | 239_a_ | 110_b_ | 79_b_ | 272_b_ | <.001 |
|  |  | % | 89,0% | 89,5% | 79,7% | 72,5% | 78,4% |  |
|  | Yes | N | 36_a_ | 28_a_ | 28_b_ | 30_b_ | 75_b_ |  |
|  |  | % | 11,0% | 10,5% | 20,3% | 27,5% | 21,6% |  |
| Notes. Subscript letters indicate if diagnostic groups differ from each other on the .05 level. The same letter means there is no statistical difference, a different letter means the groups differ from each other. | | | | | | | | |

|  | | | **PBC** | **PSC** | **AIH-PBC** | **AIH-PSC** | **AIH** | ***p*** |
| --- | --- | --- | --- | --- | --- | --- | --- | --- |
| **Comorbidities** | No | N | 135_a, b_ | 98_c_ | 71_b_ | 40_a, c_ | 194_d_ | <.001 |
|  |  | % | 40,7% | 32,6% | 45,5% | 33,6% | 56,9% |  |
|  | Yes | N | 197_a, b_ | 203_c_ | 85_b_ | 79_a, c_ | 147_d_ |  |
|  |  | % | 59,3% | 67,4% | 54,5% | 66,4% | 43,1% |  |
| Notes. Subscript letters indicate if diagnostic groups differ from each other on the .05 level. The same letter means there is no statistical difference, a different letter means the groups differ from each other. | | | | | | | | |

|  | | | **PBC** | **PSC** | **AIH-PBC** | **AIH-PSC** | **AIH** | ***p*** |
| --- | --- | --- | --- | --- | --- | --- | --- | --- |
| **Corticosteroid treatment** | No | N | 334_a_ | 294_a_ | 107_b_ | 65_c_ | 198_c_ | <.001 |
|  |  | % | 97,7% | 96,7% | 66,9% | 53,7% | 57,4% |  |
|  | Yes | N | 8_a_ | 10_a_ | 53_b_ | 56_c_ | 147_c_ |  |
|  |  | % | 2,3% | 3,3% | 33,1% | 46,3% | 42,6% |  |
| Notes. Subscript letters indicate if diagnostic groups differ from each other on the .05 level. The same letter means there is no statistical difference, a different letter means the groups differ from each other. | | | | | | | | |

**Table S3. Statistical comparisons of diagnostic groups for variables in Table 2.**

| **Dependent variable** | **Diagnostic group** | **Diagnostic group** | **Mean difference** | **Standard error** | **Sig.** | **95% Confidence interval** | |
| --- | --- | --- | --- | --- | --- | --- | --- |
|  |  |  |  |  |  | **Lower** | **Upper** |
| **Physical HRQOL (SF-12)** | PBC | PSC | -5,91417* | ,78887 | <,001 | -8,0725 | -3,7559 |
|  |  | PBC-AIH | 1,40687 | 1,10064 | ,705 | -1,6137 | 4,4274 |
|  |  | PSC-AIH | -4,47385* | 1,10569 | <,001 | -7,5143 | -1,4334 |
|  |  | AIH | -3,19932* | ,84188 | ,001 | -5,5022 | -,8964 |
|  | PSC | PBC-AIH | 7,32105* | 1,03824 | <,001 | 4,4683 | 10,1738 |
|  |  | PSC-AIH | 1,44032 | 1,04359 | ,641 | -1,4344 | 4,3150 |
|  |  | AIH | 2,71486* | ,75848 | ,003 | ,6398 | 4,7899 |
|  | PBC-AIH | PSC-AIH | -5,88072* | 1,29545 | <,001 | -9,4387 | -2,3227 |
|  |  | AIH | -4,60619* | 1,07907 | <,001 | -7,5686 | -1,6438 |
|  | PSC-AIH | AIH | 1,27453 | 1,08422 | ,765 | -1,7085 | 4,2575 |
| **Mental HRQOL (SF-12)** | PBC | PSC | ,06342 | ,86466 | 1,000 | -2,3021 | 2,4289 |
|  |  | PBC-AIH | 1,53851 | 1,02400 | ,562 | -1,2705 | 4,3475 |
|  |  | PSC-AIH | 3,03698 | 1,12486 | ,057 | -,0573 | 6,1313 |
|  |  | AIH | ,17383 | ,85682 | 1,000 | -2,1699 | 2,5175 |
|  | PSC | PBC-AIH | 1,47509 | 1,03066 | ,608 | -1,3521 | 4,3023 |
|  |  | PSC-AIH | 2,97357 | 1,13093 | ,069 | -,1371 | 6,0842 |
|  |  | AIH | ,11041 | ,86477 | 1,000 | -2,2553 | 2,4762 |
|  | PBC-AIH | PSC-AIH | 1,49847 | 1,25696 | ,756 | -1,9553 | 4,9522 |
|  |  | AIH | -1,36468 | 1,02409 | ,671 | -4,1739 | 1,4446 |
|  | PSC-AIH | AIH | -2,86316 | 1,12495 | ,085 | -5,9577 | ,2314 |
| **Anxiety severity (GAD7)** | PBC | PSC | ,83300 | ,34256 | ,108 | -,1041 | 1,7701 |
|  |  | PBC-AIH | ,09822 | ,45536 | 1,000 | -1,1520 | 1,3484 |
|  |  | PSC-AIH | -,78329 | ,48853 | ,497 | -2,1280 | ,5615 |
|  |  | AIH | ,78615 | ,34023 | ,143 | -,1445 | 1,7168 |
|  | PSC | PBC-AIH | -,73479 | ,44989 | ,477 | -1,9703 | ,5008 |
|  |  | PSC-AIH | -1,61629* | ,48343 | ,009 | -2,9475 | -,2851 |
|  |  | AIH | -,04686 | ,33286 | 1,000 | -,9575 | ,8638 |
|  | PBC-AIH | PSC-AIH | -,88150 | ,56896 | ,531 | -2,4447 | ,6817 |
|  |  | AIH | ,68793 | ,44812 | ,541 | -,5428 | 1,9187 |
|  | PSC-AIH | AIH | 1,56943* | ,48178 | ,011 | ,2427 | 2,8962 |
| **Depression severity (PHQ-9)** | PBC | PSC | ,79987 | ,39788 | ,262 | -,2886 | 1,8884 |
|  |  | PBC-AIH | -,42226 | ,48418 | ,907 | -1,7504 | ,9059 |
|  |  | PSC-AIH | -,65093 | ,51702 | ,717 | -2,0723 | ,7704 |
|  |  | AIH | ,77578 | ,39259 | ,279 | -,2981 | 1,8497 |
|  | PSC | PBC-AIH | -1,22213 | ,47046 | ,073 | -2,5133 | ,0691 |
|  |  | PSC-AIH | -1,45081* | ,50419 | ,035 | -2,8378 | -,0638 |
|  |  | AIH | -,02410 | ,37553 | 1,000 | -1,0515 | 1,0033 |
|  | PBC-AIH | PSC-AIH | -,22867 | ,57475 | ,995 | -1,8077 | 1,3504 |
|  |  | AIH | 1,19804 | ,46600 | ,078 | -,0810 | 2,4771 |
|  | PSC-AIH | AIH | 1,42671* | ,50003 | ,038 | ,0509 | 2,8025 |
| **Somatic symptom severity (SSS-8)** | PBC | PSC | 2,57132* | ,47101 | <,001 | 1,2828 | 3,8599 |
|  |  | PBC-AIH | -,31096 | ,56651 | ,982 | -1,8641 | 1,2422 |
|  |  | PSC-AIH | 1,39542 | ,61907 | ,164 | -,3057 | 3,0966 |
|  |  | AIH | 1,50901* | ,47667 | ,014 | ,2052 | 2,8128 |
|  | PSC | PBC-AIH | -2,88228* | ,53519 | <,001 | -4,3508 | -1,4138 |
|  |  | PSC-AIH | -1,17590 | ,59055 | ,274 | -2,8007 | ,4489 |
|  |  | AIH | -1,06232 | ,43900 | ,111 | -2,2632 | ,1386 |
|  | PBC-AIH | PSC-AIH | 1,70638 | ,66920 | ,083 | -,1320 | 3,5448 |
|  |  | AIH | 1,81996* | ,54018 | ,007 | ,3381 | 3,3018 |
|  | PSC-AIH | AIH | ,11359 | ,59507 | 1,000 | -1,5232 | 1,7504 |
| **Psychological burden by somatic symptoms (SSD-12)** | PBC | PSC | ,677 | ,854 | ,933 | -1,66 | 3,01 |
|  |  | PBC-AIH | -,857 | ,997 | ,911 | -3,59 | 1,88 |
|  |  | PSC-AIH | -,695 | 1,065 | ,966 | -3,62 | 2,23 |
|  |  | AIH | 1,222 | ,820 | ,569 | -1,02 | 3,46 |
|  | PSC | PBC-AIH | -1,534 | ,992 | ,534 | -4,26 | 1,19 |
|  |  | PSC-AIH | -1,372 | 1,061 | ,696 | -4,29 | 1,55 |
|  |  | AIH | ,545 | ,814 | ,963 | -1,68 | 2,77 |
|  | PBC-AIH | PSC-AIH | ,162 | 1,179 | 1,000 | -3,08 | 3,40 |
|  |  | AIH | 2,079 | ,963 | ,199 | -,57 | 4,72 |
|  | PSC-AIH | AIH | 1,917 | 1,034 | ,346 | -,93 | 4,76 |
| **Fatigue (MFIS)** | PBC | PSC | 6,238* | 1,590 | <,001 | 1,89 | 10,59 |
|  |  | PBC-AIH | -3,269 | 1,984 | ,468 | -8,72 | 2,18 |
|  |  | PSC-AIH | ,740 | 1,941 | ,996 | -4,60 | 6,07 |
|  |  | AIH | 4,393* | 1,563 | ,041 | ,12 | 8,67 |
|  | PSC | PBC-AIH | -9,507* | 1,909 | <,001 | -14,75 | -4,26 |
|  |  | PSC-AIH | -5,498* | 1,865 | ,029 | -10,63 | -,37 |
|  |  | AIH | -1,845 | 1,467 | ,717 | -5,86 | 2,17 |
|  | PBC-AIH | PSC-AIH | 4,009 | 2,210 | ,368 | -2,07 | 10,08 |
|  |  | AIH | 7,662* | 1,887 | <,001 | 2,48 | 12,85 |
|  | PSC-AIH | AIH | 3,653 | 1,842 | ,278 | -1,41 | 8,72 |
| **Physical functioning (SF-12)** | PBC | PSC | -7,04483* | ,78031 | <,001 | -9,1796 | -4,9100 |
|  |  | PBC-AIH | ,05046 | 1,08371 | 1,000 | -2,9231 | 3,0240 |
|  |  | PSC-AIH | -6,17821* | 1,00871 | <,001 | -8,9490 | -3,4074 |
|  |  | AIH | -3,78068* | ,84541 | <,001 | -6,0930 | -1,4683 |
|  | PSC | PBC-AIH | 7,09529* | 1,01278 | <,001 | 4,3125 | 9,8781 |
|  |  | PSC-AIH | ,86662 | ,93209 | ,885 | -1,6988 | 3,4320 |
|  |  | AIH | 3,26414* | ,75234 | <,001 | 1,2060 | 5,3223 |
|  | PBC-AIH | PSC-AIH | -6,22867* | 1,19764 | <,001 | -9,5175 | -2,9399 |
|  |  | AIH | -3,83114* | 1,06375 | ,003 | -6,7508 | -,9115 |
|  | PSC-AIH | AIH | 2,39753 | ,98723 | ,111 | -,3154 | 5,1105 |
| **Role physical (SF-12)** | PBC | PSC | -4,51293* | ,83501 | <,001 | -6,7972 | -2,2286 |
|  |  | PBC-AIH | 1,65395 | 1,07841 | ,541 | -1,3053 | 4,6132 |
|  |  | PSC-AIH | -,68067 | 1,14703 | ,976 | -3,8361 | 2,4747 |
|  |  | AIH | -2,31840 | ,87008 | ,060 | -4,6983 | ,0615 |
|  | PSC | PBC-AIH | 6,16688* | 1,05535 | <,001 | 3,2696 | 9,0641 |
|  |  | PSC-AIH | 3,83226* | 1,12537 | ,007 | ,7346 | 6,9299 |
|  |  | AIH | 2,19453 | ,84133 | ,070 | -,1071 | 4,4961 |
|  | PBC-AIH | PSC-AIH | -2,33462 | 1,31612 | ,391 | -5,9501 | 1,2809 |
|  |  | AIH | -3,97235* | 1,08331 | ,003 | -6,9448 | -,9998 |
|  | PSC-AIH | AIH | -1,63773 | 1,15163 | ,614 | -4,8055 | 1,5300 |
| **Bodily pain (SF-12)** | PBC | PSC | -3,88429* | ,88857 | <,001 | -6,3152 | -1,4534 |
|  |  | PBC-AIH | 2,42594 | 1,23116 | ,283 | -,9534 | 5,8053 |
|  |  | PSC-AIH | -2,23091 | 1,25894 | ,393 | -5,6944 | 1,2326 |
|  |  | AIH | -,98875 | ,91792 | ,818 | -3,4995 | 1,5220 |
|  | PSC | PBC-AIH | 6,31023* | 1,18969 | <,001 | 3,0423 | 9,5782 |
|  |  | PSC-AIH | 1,65339 | 1,21841 | ,656 | -1,7020 | 5,0088 |
|  |  | AIH | 2,89554* | ,86150 | ,007 | ,5388 | 5,2522 |
|  | PBC-AIH | PSC-AIH | -4,65684* | 1,48686 | ,016 | -8,7406 | -,5731 |
|  |  | AIH | -3,41468* | 1,21177 | ,041 | -6,7418 | -,0875 |
|  | PSC-AIH | AIH | 1,24216 | 1,23998 | ,854 | -2,1706 | 4,6550 |
| **General health (SF-12)** | PBC | PSC | -3,21697* | ,83443 | ,001 | -5,4997 | -,9342 |
|  |  | PBC-AIH | 1,75679 | 1,06937 | ,471 | -1,1782 | 4,6918 |
|  |  | PSC-AIH | -1,99084 | 1,16063 | ,427 | -5,1862 | 1,2046 |
|  |  | AIH | -2,67154* | ,81484 | ,010 | -4,9004 | -,4427 |
|  | PSC | PBC-AIH | 4,97376* | 1,07381 | <,001 | 2,0266 | 7,9209 |
|  |  | PSC-AIH | 1,22614 | 1,16472 | ,830 | -1,9803 | 4,4326 |
|  |  | AIH | ,54544 | ,82066 | ,964 | -1,6996 | 2,7905 |
|  | PBC-AIH | PSC-AIH | -3,74762* | 1,34308 | ,045 | -7,4379 | -,0574 |
|  |  | AIH | -4,42832* | 1,05866 | <,001 | -7,3345 | -1,5222 |
|  | PSC-AIH | AIH | -,68070 | 1,15077 | ,976 | -3,8498 | 2,4884 |
| **Vitality (SF-12)** | PBC | PSC | -1,73654 | ,81765 | ,211 | -3,9735 | ,5004 |
|  |  | PBC-AIH | 1,33595 | 1,08019 | ,730 | -1,6289 | 4,3008 |
|  |  | PSC-AIH | ,06275 | 1,14006 | 1,000 | -3,0745 | 3,2000 |
|  |  | AIH | -1,21417 | ,83126 | ,589 | -3,4881 | 1,0597 |
|  | PSC | PBC-AIH | 3,07249* | 1,05750 | ,032 | ,1687 | 5,9763 |
|  |  | PSC-AIH | 1,79929 | 1,11858 | ,494 | -1,2807 | 4,8793 |
|  |  | AIH | ,52237 | ,80156 | ,966 | -1,6704 | 2,7152 |
|  | PBC-AIH | PSC-AIH | -1,27320 | 1,32269 | ,872 | -4,9070 | 2,3606 |
|  |  | AIH | -2,55012 | 1,06807 | ,122 | -5,4822 | ,3820 |
|  | PSC-AIH | AIH | -1,27692 | 1,12857 | ,790 | -4,3834 | 1,8296 |
| **Social functioning (SF-12)** | PBC | PSC | -1,64025 | ,87333 | ,330 | -4,0293 | ,7489 |
|  |  | PBC-AIH | 3,08074 | 1,15524 | ,062 | -,0904 | 6,2519 |
|  |  | PSC-AIH | ,54860 | 1,17572 | ,990 | -2,6860 | 3,7832 |
|  |  | AIH | ,00207 | ,87247 | 1,000 | -2,3843 | 2,3884 |
|  | PSC | PBC-AIH | 4,72099* | 1,15080 | <,001 | 1,5617 | 7,8803 |
|  |  | PSC-AIH | 2,18885 | 1,17135 | ,338 | -1,0342 | 5,4119 |
|  |  | AIH | 1,64231 | ,86658 | ,321 | -,7283 | 4,0129 |
|  | PBC-AIH | PSC-AIH | -2,53214 | 1,39425 | ,366 | -6,3615 | 1,2972 |
|  |  | AIH | -3,07868 | 1,15014 | ,060 | -6,2361 | ,0787 |
|  | PSC-AIH | AIH | -,54654 | 1,17071 | ,990 | -3,7677 | 2,6746 |
| **Role emotional (SF-12)** | PBC | PSC | -2,54166 | ,95795 | ,062 | -5,1623 | ,0790 |
|  |  | PBC-AIH | 1,25876 | 1,18372 | ,825 | -1,9885 | 4,5060 |
|  |  | PSC-AIH | ,17176 | 1,27678 | 1,000 | -3,3397 | 3,6832 |
|  |  | AIH | -1,31374 | ,97188 | ,659 | -3,9721 | 1,3446 |
|  | PSC | PBC-AIH | 3,80042* | 1,16102 | ,010 | ,6143 | 6,9866 |
|  |  | PSC-AIH | 2,71342 | 1,25576 | ,199 | -,7419 | 6,1687 |
|  |  | AIH | 1,22792 | ,94410 | ,691 | -1,3547 | 3,8106 |
|  | PBC-AIH | PSC-AIH | -1,08700 | 1,43543 | ,942 | -5,0306 | 2,8566 |
|  |  | AIH | -2,57250 | 1,17254 | ,185 | -5,7895 | ,6445 |
|  | PSC-AIH | AIH | -1,48550 | 1,26641 | ,767 | -4,9692 | 1,9981 |
| **Mental health (SF-12)** | PBC | PSC | -,99425 | ,80213 | ,728 | -3,1886 | 1,2001 |
|  |  | PBC-AIH | 1,19562 | ,99465 | ,750 | -1,5337 | 3,9250 |
|  |  | PSC-AIH | 2,23377 | ,99781 | ,169 | -,5095 | 4,9771 |
|  |  | AIH | -,68945 | ,79890 | ,910 | -2,8746 | 1,4957 |
|  | PSC | PBC-AIH | 2,18988 | 1,00211 | ,188 | -,5597 | 4,9395 |
|  |  | PSC-AIH | 3,22802* | 1,00525 | ,013 | ,4646 | 5,9914 |
|  |  | AIH | ,30480 | ,80817 | ,996 | -1,9060 | 2,5156 |
|  | PBC-AIH | PSC-AIH | 1,03815 | 1,16466 | ,900 | -2,1605 | 4,2368 |
|  |  | AIH | -1,88507 | ,99952 | ,327 | -4,6275 | ,8574 |
|  | PSC-AIH | AIH | -2,92322* | 1,00267 | ,032 | -5,6795 | -,1669 |
| Notes. *Indicates significant mean difference (p<0.05). | | | | | | | |

1. **DATA TABLE FOR FIGURE 1**

**Table S4.** Proportions of patients screening positive for an anxiety or a depressive disorder by diagnostic group.

| **Variable** | ***N*_missing_** | **PBC** | **PBC-AIH** | **PSC** | **PSC-AIH** | **AIH** | **χ^2^ (4)** | ***p*** |
| --- | --- | --- | --- | --- | --- | --- | --- | --- |
| **Screened positive for anxiety disorder (GAD-7 ≥10)** | 41 | 48 (15%)_a_ | 28 (18%)_a,b_ | 36 (12%)_a_ | 27 (23%)_b_ | 43 (13%)_a_ | 11.0 | .027 |
| yes |  |  |  |  |  |  |  |  |
| **Screened positive for depression (PHQ-9 ≥10)** | 43 | 64 (20%)_a,b,c_ | 37 (24%)_b_ | 44 (15%)_c_ | 27 (23%)_a,b_ | 53 (16%)_a,c_ | 9.5 | .049 |
| yes |  |  |  |  |  |  |  |  |

*Notes.* Subscript letters indicate the results of the post-hoc procedures. If two values have a different letter, this means they differ from each other at the .05 level, while the same letter means there is no statistical difference.

1. **DETAILED RESULTS OF THE REGRESSION MODELS**

**Table S5**

Detailed results of the hierarchical multiple regression analysis for physical HRQOL in patients with PBC & PBC-AIH.

| **Variable** | **Block 1** | | | **Block 2** | | | | **Block 3** | | | **Block 4** | | | **Block 5** | | | |
| --- | --- | --- | --- | --- | --- | --- | --- | --- | --- | --- | --- | --- | --- | --- | --- | --- | --- |
|  | *B* | *SE(B)* | *p* | *B* | *SE(B)* | *p* | | *B* | *SE(B)* | *p* | *B* | *SE(B)* | *p* | | *B* | *SE(B)* | *p* |
| **(Intercept)** | 50.32 | 3.60 | <.001 | 51.56 | 3.66 | <.001 | | 51.60 | 3.66 | <.001 | 64.81 | 2.88 | <.001 | | **64.20** | 2.93 | <.001 |
| **Age** | -0.12 | 0.05 | .023 | -0.12 | 0.05 | .015 | | -0.12 | 0.05 | .019 | -0.21 | 0.04 | <.001 | | **-0.20** | 0.04 | <.001 |
| Gender | -0.05 | 2.03 | .981 | -0.16 | 2.02 | .938 | | -0.25 | 2.03 | .903 | 0.59 | 1.54 | .701 | | 0.50 | 1.54 | .743 |
| PBC-AIH variant syndrome^1^ |  |  |  | -2.24 | 1.28 | .081 | | -2.07 | 1.30 | .112 | -1.44 | 0.98 | .144 | | -1.37 | 0.99 | .164 |
| Cirrhosis |  |  |  |  |  |  | | -1.55 | 1.69 | .358 | -1.71 | 1.28 | .181 | | -1.67 | 1.28 | .192 |
| **Fatigue** |  |  |  |  |  |  | |  |  |  | -0.38 | 0.02 | <.001 | | **-0.42** | 0.04 | <.001 |
| Depression severity |  |  |  |  |  |  | |  |  |  |  |  |  | | 0.18 | 0.15 | .223 |
| *R^2^* | .014 | | | .022 | | | .024 | | | | .441 | | | | .444 | | |
| Δ*R^2^* | .014 | | | .008 | | | .002 | | | | .418 | | | | .002 | | |
| *F* | 2.62 | | | 3.05 | | | 0.85 | | | | 281.01 | | | | 1.49 | | |
| *p* (Δ*F)* | .074 | | | .081 | | | .358 | | | | <.001 | | | | .223 | | |

*Notes*. *N*=382 ^1^reference category = PBC; significant variables in the final model are shown in bold.

**Table S6**

Detailed results of the hierarchical multiple regression analysis for mental HRQOL in patients with PBC & PBC-AIH.

| **Variable** | **Block 1** | | | **Block 2** | | | | **Block 3** | | | **Block 4** | | | **Block 5** | | | |
| --- | --- | --- | --- | --- | --- | --- | --- | --- | --- | --- | --- | --- | --- | --- | --- | --- | --- |
|  | *B* | *SE(B)* | *p* | *B* | *SE(B)* | *p* | | *B* | *SE(B)* | *p* | *B* | *SE(B)* | *p* | | *B* | *SE(B)* | *p* |
| **(Intercept)** | 36.26 | 3.30 | <.001 | 36.59 | 3.37 | <.001 | | 36.60 | 3.37 | <.001 | 47.97 | 2.78 | <.001 | | **51.74** | 2.59 | <.001 |
| **Age** | 0.21 | 0.05 | <.001 | 0.21 | 0.05 | <.001 | | 0.21 | 0.05 | <.001 | 0.14 | 0.04 | <.001 | | **0.09** | 0.04 | .015 |
| Gender | -1.57 | 1.86 | .398 | -1.60 | 1.86 | .390 | | -1.62 | 1.86 | .385 | -0.90 | 1.48 | .542 | | -0.37 | 1.36 | .786 |
| PBC-AIH variant syndrome^1^ |  |  |  | -0.60 | 1.18 | .609 | | -0.56 | 1.20 | .639 | -0.02 | 0.95 | .981 | | -0.44 | 0.87 | .618 |
| Cirrhosis |  |  |  |  |  |  | | -0.38 | 1.55 | .806 | -0.52 | 1.23 | .672 | | -0.78 | 1.13 | .489 |
| **Fatigue** |  |  |  |  |  |  | |  |  |  | -0.33 | 0.02 | <.001 | | **-0.11** | 0.03 | .001 |
| **Depression severity** |  |  |  |  |  |  | |  |  |  |  |  |  | | **-1.12** | 0.13 | <.001 |
| *R^2^* | .052 | | | .053 | | | .053 | | | | .407 | | | | .501 | | |
| Δ*R^2^* | .052 | | | .001 | | | .000 | | | | .354 | | | | .095 | | |
| *F (df1, df2)* | 10.47 | | | 0.26 | | | 0.06 | | | | 224.02 | | | | 71.13 | | |
| *p* (Δ*F)* | <.001 | | | .609 | | | .809 | | | | <.001 | | | | <.001 | | |

*Notes*. *N*=382, ^1^reference category = PBC; significant variables in the final model are shown in bold.

**Table S7**

Detailed results of the hierarchical multiple regression analysis for physical HRQOL in patients with PSC & PSC-AIH.

| **Variable** | **Block 1** | | | **Block 2** | | | | **Block 3** | | | **Block 4** | | | **Block 5** | | | |
| --- | --- | --- | --- | --- | --- | --- | --- | --- | --- | --- | --- | --- | --- | --- | --- | --- | --- |
|  | *B* | *SE(B)* | *p* | *B* | *SE(B)* | *p* | | *B* | *SE(B)* | *p* | *B* | *SE(B)* | *p* | | *B* | *SE(B)* | *p* |
| **(Intercept)** | 54.32 | 1.47 | <.001 | 55.76 | 1.62 | <.001 | | 55.79 | 1.62 | <.001 | 59.63 | 1.38 | <.001 | | **59.48** | 1.43 | <.001 |
| **Age** | -0.10 | 0.03 | .005 | -0.12 | 0.04 | .001 | | -0.11 | 0.04 | .001 | -0.11 | 0.03 | <.001 | | **-0.11** | 0.03 | <.001 |
| Gender | -2.66 | 0.99 | .008 | -2.58 | 0.99 | .010 | | -2.58 | 0.99 | .010 | -1.43 | 0.83 | .086 | | -1.44 | 0.83 | .084 |
| PSC-AIH variant syndrome^1^ |  |  |  | -2.25 | 1.10 | .041 | | -2.01 | 1.13 | .075 | -0.14 | 0.95 | .884 | | -0.14 | 0.95 | .880 |
| Cirrhosis |  |  |  |  |  |  | | -1.26 | 1.38 | .361 | -0.85 | 1.15 | .457 | | -0.81 | 1.15 | .482 |
| **Fatigue** |  |  |  |  |  |  | |  |  |  | -0.29 | 0.02 | <.001 | | **-0.30** | 0.04 | <.001 |
| Depression severity |  |  |  |  |  |  | |  |  |  |  |  |  | | 0.06 | 0.14 | .679 |
| *R^2^* | .054 | | | .066 | | | .068 | | | | .357 | | | | .357 | | |
| Δ*R^2^* | .054 | | | .012 | | | .002 | | | | .288 | | | | .000 | | |
| *F* | 9.73 | | | 4.22 | | | 0.84 | | | | 150.51 | | | | 0.17 | | |
| *p* (Δ*F)* | <.001 | | | .041 | | | .361 | | | | <.001 | | | | .679 | | |

*Notes*. *N*=342, ^1^reference category = PSC, significant variables in the final model are shown in bold.

**Table S8**

Detailed results of the hierarchical multiple regression analysis for mental HRQOL in patients with PSC & PSC-AIH

| **Variable** | **Block 1** | | | **Block 2** | | | | **Block 3** | | | **Block 4** | | | **Block 5** | | | |
| --- | --- | --- | --- | --- | --- | --- | --- | --- | --- | --- | --- | --- | --- | --- | --- | --- | --- |
|  | *B* | *SE(B)* | *p* | *B* | *SE(B)* | *p* | | *B* | *SE(B)* | *p* | *B* | *SE(B)* | *p* | | *B* | *SE(B)* | *p* |
| (Intercept) | 43.30 | 1.75 | <.001 | 45.25 | 1.93 | <.001 | | 45.21 | 1.93 | <.001 | 50.70 | 1.48 | <.001 | | 53.82 | 1.38 | <.001 |
| Age | 0.11 | 0.04 | .009 | 0.08 | 0.04 | .058 | | 0.08 | 0.04 | .067 | .09 | 0.03 | .007 | | 0.05 | 0.03 | .069 |
| Gender | -2.18 | 1.19 | .068 | -2.07 | 1.18 | .082 | | -2.06 | 1.18 | .083 | -0.41 | 0.89 | .645 | | -0.23 | 0.80 | .774 |
| PSC-AIH variant syndrome^1^ |  |  |  | -3.07 | 1.31 | .020 | | -3.34 | 1.35 | .014 | -0.66 | 1.02 | .518 | | -0.56 | 0.92 | .538 |
| Cirrhosis |  |  |  |  |  |  | | 1.41 | 1.64 | .392 | 1.99 | 1.23 | .107 | | 1.10 | 1.11 | .323 |
| **Fatigue** |  |  |  |  |  |  | |  |  |  | -0.41 | 0.03 | <.001 | | **-0.14** | 0.04 | <.001 |
| **Depression severity** |  |  |  |  |  |  | |  |  |  |  |  |  | | **-1.25** | 0.14 | <.001 |
| *R^2^* | .024 | | | .040 | | | .042 | | | | .467 | | | | .570 | | |
| Δ*R^2^* | .024 | | | .016 | | | .002 | | | | .425 | | | | .103 | | |
| *F (df1, df2)* | 4.24 | | | 5.49 | | | 0.73 | | | | 267.88 | | | | 80.51 | | |
| *p* (Δ*F)* | .015 | | | .020 | | | .392 | | | | <.001 | | | | <.001 | | |

*Notes*. *N*=342, ^1^reference category = PSC; significant variables in the final model are shown in bold.

1. **ADDITIONAL EXPLORATORY ANALYSIS FOR AUTOIMMUNE HEPATATIS AND VARIANT SYNDROMES**

***HRQOL in patients with AIH and PBC-AIH.***

*Physical QoL.* In patients with AIH and PBC-AIH, the block with gender and age significantly explained variation in physical HRQOL (Δ*R*^2^=.107, *p*<.001). Having a PBC-AIH variant syndrome significantly added the degree of explained variation (Δ*R*^2^=.019, *p*=.003). Adding cirrhosis further improved the model (Δ*R*^2^=.020, *p*=.002), as did fatigue (Δ*R*^2^=.229, *p*<.001). Depression severity did not improve the model (Δ*R*^2^=.000, *p*=.862). Significant predictors in the final model were age (*B*=-.153, *p*<.001), fatigue (*B*=-.302, *p*<.001) and cirrhosis (*B*=-2.681, *p*=.012). The detailed results are displayed in **table S9.**

**Table S9**

Detailed results of the hierarchical multiple regression for physical HRQOL in patients with AIH & PBC-AIH.

| **Variable** | **Block 1** | | | **Block 2** | | | | **Block 3** | | | **Block 4** | | | **Block 5** | | | |
| --- | --- | --- | --- | --- | --- | --- | --- | --- | --- | --- | --- | --- | --- | --- | --- | --- | --- |
|  | *B* | *SE(B)* | *p* | *B* | *SE(B)* | *p* | | *B* | *SE(B)* | *p* | *B* | *SE(B)* | *p* | | *B* | *SE(B)* | *p* |
| **(Intercept)** | 58.37 | 1.91 | <.001 | 57.84 | 1.90 | <.001 | | 58.33 | 1.88 | <.001 | 62.24 | 1.64 | <.001 | | **62.17** | 1.69 | <.001 |
| **Age** | -0.18 | 0.03 | <.001 | -0.16 | 0.03 | <.001 | | -0.15 | 0.03 | <.001 | -0.15 | 0.03 | <.001 | | **-0.15** | 0.03 | <.001 |
| Gender | -4.16 | 1.25 | .001 | -3.59 | 1.26 | .004 | | -3.72 | 1.24 | .003 | -1.63 | 1.08 | .132 | | -1.64 | 1.08 | .131 |
| PBC-AIH variant syndrome^1^ |  |  |  | -3.49 | 1.18 | .003 | | -3.48 | 1.17 | .003 | -1.97 | 1.01 | .051 | | -1.96 | 1.01 | .053 |
| **Cirrhosis** |  |  |  |  |  |  | | -3.82 | 1.24 | .002 | -2.69 | 1.07 | .012 | | -**2.68** | 1.07 | .012 |
| **Fatigue** |  |  |  |  |  |  | |  |  |  | -0.30 | 0.03 | <.001 | | **-0.30** | 0.04 | <.001 |
| Depression severity |  |  |  |  |  |  | |  |  |  |  |  |  | | 0.03 | 0.15 | .862 |
| *R^2^* | .107 | | | .126 | | | .146 | | | | .376 | | | | .376 | | |
| Δ*R^2^* | .107 | | | .019 | | | .020 | | | | .229 | | | | .000 | | |
| *F* | 23.84 | | | 8.79 | | | 9.51 | | | | 145.84 | | | | .030 | | |
| *p* (Δ*F)* | <.001 | | | .003 | | | .002 | | | | <.001 | | | | .862 | | |

*Notes*. *N*=403, ^1^reference category = AIH; significant variables in the final model are shown in bold.

*Mental QoL*. In patients with AIH and PBC-AIH, the block with gender and age significantly contributed to the model (Δ*R*^2^=.039, *p*<.001), but being affected by a variant syndrome did not explain any variation of mental HRQOL (Δ*R*^2^=.002, *p*=.315). Cirrhosis did not improve the model, whereas fatigue and depression severity did. In the final model, age (*B*=.070, *p*=.004), depression severity (*B*=-1.032, *p*<.001) and fatigue (*B*=-.151, *p*<.001) were significant predictors of mental HRQOL. The regression table can be found in **table S10.**

**Table S10**

Detailed results of the hierarchical multiple regression analysis for mental HRQOL in patients with AIH & PBC-AIH

| **Variable** | **Block 1** | | | **Block 2** | | | | **Block 3** | | | **Block 4** | | | **Block 5** | | | |
| --- | --- | --- | --- | --- | --- | --- | --- | --- | --- | --- | --- | --- | --- | --- | --- | --- | --- |
|  | *B* | *SE(B)* | *p* | *B* | *SE(B)* | *p* | | *B* | *SE(B)* | *p* | *B* | *SE(B)* | *p* | | *B* | *SE(B)* | *p* |
| (Intercept) | 44.86 | 1.94 | <.001 | 44.67 | 1.94 | <.001 | | 44.76 | 1.95 | <.001 | 49.64 | 1.56 | <.001 | | 52.25 | 1.50 | <.001 |
| **Age** | 0.10 | 0.03 | .002 | 0.11 | 0.03 | <.001 | | 0.11 | 0.03 | <.001 | 0.11 | 0.03 | <.001 | | **0.07** | 0.03 | .004 |
| Gender | -3.47 | 1.27 | .007 | -3.28 | 1.29 | .011 | | -3.30 | 1.29 | .011 | -0.73 | 1.02 | .476 | | -0.40 | 0.96 | .677 |
| PBC-AIH variant syndrome^1^ |  |  |  | -1.21 | 1.21 | .315 | | -1.22 | 1.21 | .315 | 0.70 | 0.95 | .462 | | 0.40 | 0.89 | .656 |
| Cirrhosis |  |  |  |  |  |  | | -0.74 | 1.28 | .563 | 0.56 | 1.01 | .576 | | 0.25 | 0.94 | .794 |
| **Fatigue** |  |  |  |  |  |  | |  |  |  | -0.37 | 0.02 | <.001 | | **-0.15** | 0.04 | <.001 |
| **Depression severity** |  |  |  |  |  |  | |  |  |  |  |  |  | | **-1.03** | 0.14 | <.001 |
| *R^2^* | .039 | | | .041 | | | .042 | | | | .413 | | | | .487 | | |
| Δ*R^2^* | .039 | | | .002 | | | .001 | | | | .371 | | | | .074 | | |
| *F* | 8.06 | | | 1.01 | | | .034 | | | | 252.01 | | | | 57.13 | | |
| *p* (Δ*F)* | <.001 | | | .315 | | | .563 | | | | <.001 | | | | <.001 | | |

*Notes*. *N*=404, ^1^reference category = AIH; significant variables in the final model are shown in bold.

***HRQOL in patients with AIH and PSC-AIH.***

*Physical HRQOL.* In patients with AIH and PSC-AIH, gender and age significantly explained variation in physical HRQOL (Δ*R*^2^=.109, *p*<.001). The variant syndrome added a significant degree of explained variation (Δ*R*^2^=.010, *p*=.037). Adding cirrhosis further improved the model, as did fatigue. Depression severity added no increase in explained variance. Significant predictors in the final model were age (*B*=-.159, *p*<.001), fatigue (*B*=-.280, *p*<.001) and cirrhosis (*B*=-2.371, *p*=.019). Details of the results are in **table S11.**

**Table S11**

Detailed results of the hierarchical multiple regression analysis for physical HRQOL in patients with AIH & PSC-AIH.

| **Variable** | **Block 1** | | | **Block 2** | | | | **Block 3** | | | **Block 4** | | | **Block 5** | | | |
| --- | --- | --- | --- | --- | --- | --- | --- | --- | --- | --- | --- | --- | --- | --- | --- | --- | --- |
|  | *B* | *SE(B)* | *p* | *B* | *SE(B)* | *p* | | *B* | *SE(B)* | *p* | *B* | *SE(B)* | *p* | | *B* | *SE(B)* | *p* |
| **(Intercept)** | 56.73 | 1.44 | <.001 | 58.68 | 1.71 | <.001 | | 58.99 | 1.70 | <.001 | 61.89 | 1.50 | <.001 | | **61.85** | 1.54 | <.001 |
| **Age** | -0.16 | 0.03 | <.001 | -0.18 | 0.03 | <.001 | | -0.17 | 0.03 | <.001 | -0.16 | 0.03 | <.001 | | **-0.16** | 0.03 | <.001 |
| Gender | -2.77 | 1.05 | .008 | -3.33 | 1.08 | .002 | | -3.34 | 1.07 | .002 | -1.39 | 0.94 | .140 | | -1.39 | 0.94 | .140 |
| PSC-AIH variant syndrome^1^ |  |  |  | -2.58 | 1.23 | .037 | | -2.24 | 1.23 | .069 | -0.38 | 1.08 | .728 | | -0.38 | 1.08 | .725 |
| **Cirrhosis** |  |  |  |  |  |  | | -3.36 | 1.15 | .004 | -2.38 | 1.01 | .019 | | **-2.37** | 1.01 | .019 |
| **Fatigue** |  |  |  |  |  |  | |  |  |  | -0.28 | 0.03 | <.001 | | **-0.28** | 0.04 | <.001 |
| Depression severity |  |  |  |  |  |  | |  |  |  |  |  |  | | 0.01 | 0.14 | .921 |
| *R^2^* | .109 | | | .119 | | | .138 | | | | .352 | | | | .352 | | |
| Δ*R^2^* | .109 | | | .010 | | | .019 | | | | .214 | | | | .000 | | |
| *F* | 23.45 | | | 4.37 | | | 8.48 | | | | 126.02 | | | | .010 | | |
| *p* (Δ*F)* | <.001 | | | .037 | | | .004 | | | | <.001 | | | | .921 | | |

*Notes*. *N*=388, ^1^reference category = AIH; significant variables in the final model are shown in bold.

*Mental Qol.* In patients with AIH and PSC-AIH, the block with gender and age significantly contributed to the regression model (Δ*R*^2^=.044, *p*<.001). Having a variant syndrome significantly contributed to the model (Δ*R*^2^=.013, *p*=.024). The third block with cirrhosis did not contribute to explained variance while fatigue and depression severity further improved the model. Significant predictors in the final model were age (*B*=.083, *p*=.001), depression severity (*B*=-1.100, *p*<.001) and fatigue (*B*=-.158, *p*<.001). Detailed results can be found in **table S12**.

**Table S12**

Detailed results of the hierarchical multiple regression analysis for mental HRQOL in patients with AIH & PSC-AIH.

| **Variable** | **Block 1** | | | **Block 2** | | | | **Block 3** | | | **Block 4** | | | **Block 5** | | | |
| --- | --- | --- | --- | --- | --- | --- | --- | --- | --- | --- | --- | --- | --- | --- | --- | --- | --- |
|  | *B* | *SE(B)* | *p* | *B* | *SE(B)* | *p* | | *B* | *SE(B)* | *p* | *B* | *SE(B)* | *p* | | *B* | *SE(B)* | *p* |
| **(Intercept)** | 42.64 | 1.59 | <.001 | 44.97 | 1.88 | <.001 | | 44.97 | 1.89 | <.001 | 49.09 | 1.48 | <.001 | | **51.95** | 1.40 | <.001 |
| **Age** | 0.13 | 0.03 | <.001 | 0.10 | 0.03 | .002 | | 0.10 | 0.03 | .003 | 0.12 | 0.03 | <.001 | | **0.08** | 0.02 | <.001 |
| Gender | -2.54 | 1.15 | .028 | -3.21 | 1.19 | .007 | | -3.21 | 1.19 | .007 | -0.46 | 0.93 | .622 | | -0.34 | 0.86 | .688 |
| PSC-AIH variant syndrome^1^ |  |  |  | -3.08 | 1.36 | .024 | | -3.08 | 1.37 | .024 | -0.40 | 1.07 | .710 | | -0.06 | 0.98 | .950 |
| Cirrhosis |  |  |  |  |  |  | | 0.06 | 1.28 | .965 | 1.34 | 0.99 | .177 | | 0.81 | 0.91 | .378 |
| **Fatigue** |  |  |  |  |  |  | |  |  |  | -0.40 | 0.02 | <.001 | | **-0.16** | 0.04 | <.001 |
| **Depression severity** |  |  |  |  |  |  | |  |  |  |  |  |  | | **-1.10** | 0.13 | <.001 |
| *R^2^* | .044 | | | .056 | | | .056 | | | | .440 | | | | .529 | | |
| Δ*R^2^* | .044 | | | .013 | | | .000 | | | | .384 | | | | .089 | | |
| *F* | 8.85 | | | 5.15 | | | 0.00 | | | | 262.67 | | | | 71.96 | | |
| *p* (Δ*F)* | <.001 | | | .024 | | | .965 | | | | <.001 | | | | <.001 | | |

*Notes*. *N*=389, ^1^reference category = AIH; significant variables in the final model are shown in bold.

**REFERENCES**

1. Ware J, Jr., Kosinski M, Keller SD. A 12-Item Short-Form Health Survey: construction of scales and preliminary tests of reliability and validity. *Med Care*. Mar 1996;34(3):220-33. doi:10.1097/00005650-199603000-00003

2. Gandek B, Ware JE, Aaronson NK, et al. Cross-validation of item selection and scoring for the SF-12 Health Survey in nine countries: results from the IQOLA Project. International Quality of Life Assessment. *J Clin Epidemiol*. Nov 1998;51(11):1171-8. doi:10.1016/s0895-4356(98)00109-7

3. Cheung AC, Patel H, Meza-Cardona J, Cino M, Sockalingam S, Hirschfield GM. Factors that Influence Health-Related Quality of Life in Patients with Primary Sclerosing Cholangitis. *Dig Dis Sci*. Jun 2016;61(6):1692-9. doi:10.1007/s10620-015-4013-1

4. Janik MK, Wunsch E, Raszeja-Wyszomirska J, et al. Autoimmune hepatitis exerts a profound, negative effect on health-related quality of life: A prospective, single-centre study. *Liver International*. 2019;39(1):215-221. doi:10.1111/liv.13960

5. Uhlenbusch N, Bal A, Balogh B, et al. Improving quality of life in patients with rare autoimmune liver diseases by structured peer-delivered support (Q.RARE.LI): study protocol for a transnational effectiveness-implementation hybrid trial. *BMC Psychiatry*. Mar 24 2023;23(1):193. doi:10.1186/s12888-023-04669-0

6. Fisk JD, Ritvo PG, Ross L, Haase DA, Marrie TJ, Schlech WF. Measuring the functional impact of fatigue: initial validation of the fatigue impact scale. *Clin Infect Dis*. Jan 1994;18 Suppl 1:79-83. doi:10.1093/clinids/18.supplement_1.s79

7. Sogolow ED, Lasker JN, Short LM. Fatigue as a major predictor of quality of life in women with autoimmune liver disease: the case of primary biliary cirrhosis. *Womens Health Issues*. Jul-Aug 2008;18(4):336-42. doi:10.1016/j.whi.2007.12.005

8. Kos D, Kerckhofs E, Carrea I, Verza R, Ramos M, Jansa J. Evaluation of the Modified Fatigue Impact Scale in four different European countries. *Multiple Sclerosis Journal*. 2005;11(1):76-80. doi:10.1191/1352458505ms1117oa

9. Löwe B, Spitzer RL, Gräfe K, et al. Comparative validity of three screening questionnaires for DSM-IV depressive disorders and physicians' diagnoses. *J Affect Disord*. Feb 2004;78(2):131–40. doi:10.1016/s0165-0327(02)00237-9

10. Pressler SJ, Subramanian U, Perkins SM, et al. Measuring depressive symptoms in heart failure: validity and reliability of the patient health questionnaire-8. *American journal of critical care : an official publication, American Association of Critical-Care Nurses*. Mar 2011;20(2):146–52. doi:10.4037/ajcc2010931

11. Shin C, Lee SH, Han KM, Yoon HK, Han C. Comparison of the Usefulness of the PHQ-8 and PHQ-9 for Screening for Major Depressive Disorder: Analysis of Psychiatric Outpatient Data. *Psychiatry Investig*. Apr 2019;16(4):300–5. doi:10.30773/pi.2019.02.01

12. McGuire LC, Strine TW, Allen RS, Anderson LA, Mokdad AH. The Patient Health Questionnaire 8: current depressive symptoms among U.S. older adults, 2006 Behavioral Risk Factor Surveillance System. *The American journal of geriatric psychiatry : official journal of the American Association for Geriatric Psychiatry*. Apr 2009;17(4):324–34. doi:10.1097/JGP.0b013e3181953bae

13. Kroenke K, Strine TW, Spitzer RL, Williams JB, Berry JT, Mokdad AH. The PHQ-8 as a measure of current depression in the general population. *J Affect Disord*. Apr 2009;114(1-3):163–73. doi:10.1016/j.jad.2008.06.026

14. Spitzer RL, Kroenke K, Williams JB, Löwe B. A brief measure for assessing generalized anxiety disorder: the GAD-7. *Archives of internal medicine*. May 22 2006;166(10):1092–7. doi:10.1001/archinte.166.10.1092

15. Löwe B, Decker O, Müller S, et al. Validation and standardization of the Generalized Anxiety Disorder Screener (GAD-7) in the general population. *Med Care*. Mar 2008;46(3):266–74. doi:10.1097/MLR.0b013e318160d093

16. Kroenke K, Spitzer RL, Williams JB, Monahan PO, Löwe B. Anxiety disorders in primary care: prevalence, impairment, comorbidity, and detection. *Annals of internal medicine*. Mar 6 2007;146(5):317–25. doi:10.7326/0003-4819-146-5-200703060-00004

17. Kroenke K, Spitzer RL, Williams JBW. The PHQ-15: Validity of a New Measure for Evaluating the Severity of Somatic Symptoms. *Psychosomatic Medicine*. 2002;64(2):258-66.

18. Kroenke K, Spitzer RL, Williams JB, Löwe B. The Patient Health Questionnaire Somatic, Anxiety, and Depressive Symptom Scales: a systematic review. *Gen Hosp Psychiatry*. Jul-Aug 2010;32(4):345-59. doi:10.1016/j.genhosppsych.2010.03.006

19. Gierk B, Kohlmann S, Toussaint A, et al. Assessing somatic symptom burden: A psychometric comparison of the Patient Health Questionnaire—15 (PHQ-15) and the Somatic Symptom Scale—8 (SSS-8). *Journal of Psychosomatic Research*. 2015/04/01/ 2015;78(4):352-55. doi:10.1016/j.jpsychores.2014.11.006

20. Toussaint A, Murray AM, Voigt K, et al. Development and Validation of the Somatic Symptom Disorder-B Criteria Scale (SSD-12). *Psychosom Med*. Jan 2016;78(1):5-12. doi:10.1097/psy.0000000000000240

21. Toussaint A, Riedl B, Kehrer S, Schneider A, Löwe B, Linde K. Validity of the Somatic Symptom Disorder-B Criteria Scale (SSD-12) in primary care. *Fam Pract*. May 23 2018;35(3):342-347. doi:10.1093/fampra/cmx116
